# Research on the construction of cheerleading technique evaluation and teaching system integrating deep visual recognition and cognitive feedback mechanism

**DOI:** 10.1038/s41598-025-18237-x

**Published:** 2025-09-26

**Authors:** Yao Lu, Ziyu Wang, Meijia Chen

**Affiliations:** 1 College of Sports, Sejong University, Gwangjin-gu, Seoul, 05006 South Korea; 2 Department of Physical education, The graduate School, Sangmyung University, Seoul, 03016 South Korea

**Keywords:** Deep learning, Sports coaching, Cognitive feedback, Computational models, Neurophysiology

## Abstract

This paper describes an advanced system for the evaluation and teaching of cheerleading techniques that combines deep vision perception with cognitive feedback mechanisms. The method uses spatial feature extraction via convolutional neural networks and temporal movement analysis with recurrent neural networks, along with 3D pose estimation, to facilitate automatic evaluation of the techniques. A cognitive feedback mechanism, which is multi-modal in nature and draws principles from motor learning, supplies customized teaching through the visual, auditory, and haptic pathways. The system achieves 92.4% accuracy in technique classification with real-time processing at 35.4 fps, reduces training time by 35%, and improves skill retention to 89.3% at 4 weeks post-training compared to conventional coaching methods. The paper contributes to the application of artificial intelligence technology to sports education by providing a new way of objective analysis of performance and learning adaptation in the context of cheerleading training.

## Introduction

Cheerleading is now a very specialized sport that requires the precise performance of complex motions and choreographed routines by teams. Conventional evaluation techniques rely heavily on the subjectivity of judgments from coaches and judges, leading to biased judgments and poor quality of feedback^1^. The subjectivity of manual evaluation poses great challenges in the provision of objective and reproducible performance measures, which are essential for the refinement of skills^2^. Recent developments in computer vision and deep learning technologies have revolutionized the sports analysis space with unparalleled promise for automatically evaluating sporting techniques^3^. Motion-capture and pose estimation algorithms have proved highly suitable for analysis of sportsperson performance for a large set of sporting activities^4^. Cheerleading as a sporting activity is different because the sport is a synthesis of gymnastics, air stunts, and dance which are necessarily synchronized over multiple sportspersons^5^. The absence of the immediate feedback mechanism along with the provision of the tailored coaching also discourages mastering the skills under the existing training protocols^6^.The proposed research project will bridge such a limitation by developing an intelligent mechanism integrating the new developments in computer vision technologies and cognitive feedback methodologies such that sporting techniques can be made automatic for analysis as well as learning support for individuals. Specifically, our system employs: (1) a CNN-LSTM architecture that extracts spatial-temporal features to provide objective technique classification with 92.4% accuracy, addressing subjective judgment issues; (2) real-time processing capabilities (35.4 fps) coupled with multi-modal feedback (visual AR, spatial audio, and haptic) to deliver immediate corrections; (3) a transformer-based occlusion handling module achieving 85.3% PCK on occluded joints to resolve multi-person interaction challenges; and (4) a Bayesian skill modeling framework that personalizes training paths based on individual progress, reducing time-to-proficiency by 35%. The proposed system is designed to complement the training of cheerleading by enabling objective evaluation of the performance, immediate correction for the errors, and learning pathways with adaptation as per the capability and the progress of the individuals.

The primary objectives of this research are: (1) to develop and validate a real-time cheerleading technique evaluation system achieving > 90% classification accuracy while maintaining interactive frame rates (> 30 fps) for practical training applications; (2) to design and implement a multi-modal cognitive feedback mechanism that accelerates skill acquisition by > 30% compared to traditional coaching methods; (3) to create robust algorithms for handling multi-person occlusions in complex formations, achieving > 80% pose estimation accuracy on occluded joints; and (4) to establish a personalized learning framework that adapts to individual skill levels and learning styles, validated through controlled studies with diverse athlete populations. We hypothesize that the integration of deep visual recognition with theory-grounded cognitive feedback will significantly enhance both the objectivity of performance assessment and the efficiency of skill acquisition in cheerleading training. The scope of this study encompasses individual skill development and small-group formations (2–5 athletes), with evaluation focused on fundamental to advanced techniques as defined by international cheerleading standards. While team-level choreography optimization and competition scoring prediction represent important future extensions, they remain outside the current scope to ensure thorough validation of the core technological innovations.

## Literature review

### Computer vision in sports analysis

The evolution of motion capture systems has transformed sports performance analysis from manual observation to automated digital assessment. Early marker-based systems have given way to markerless approaches utilizing deep learning architectures^7^. Convolutional neural networks have demonstrated exceptional capability in extracting spatial features from athlete movements, while recurrent networks effectively model temporal dynamics^8^. Recent advances in 3D pose estimation enable accurate reconstruction of human body configurations from monocular video streams, achieving real-time processing speeds suitable for immediate coaching feedback. OpenPose and MediaPipe frameworks have emerged as popular solutions for sports applications, offering robust keypoint detection across diverse lighting conditions and camera angles.These technological advances have facilitated the development of comprehensive performance evaluation systems across multiple sports disciplines, from swimming stroke analysis to basketball shooting form assessment, establishing a foundation for automated coaching assistance^9–11^.

However, existing computer vision approaches in sports analysis exhibit critical limitations when applied to cheerleading. Current systems focus primarily on individual athlete tracking, neglecting the synchronized multi-person interactions essential in cheerleading routines. Moreover, the computational demands of state-of-the-art models (12–15 fps) fall short of the real-time requirements for effective coaching feedback. Our proposed system addresses these gaps by implementing a specialized CNN-LSTM architecture optimized for multi-person scenarios while maintaining 35.4 fps processing speed.

### Cheerleading technique assessment

Traditional evaluation of cheerleading is based on subjective scoring by certified judges using standardized rubrics covering the performance of techniques, synchronization, and artistic expression^12^. Biomechanical studies have identified key kinematic parameters of cheerleading stunts, including joint angles, center of mass trajectories, and force distribution patterns that are important for both effectiveness and safety of performance^13^. Technical demands include the individual proficiency required in performing tumbling passes and jumps, as well as intricate group formations requiring precise spatial-temporal coordination. Prior efforts at automation have focused primarily on the recognition of individual skills using traditional computer vision methods, with limited success due to the intrinsic complexities of the sport. The multi-individual nature of cheerleading stunts, rapid transitions between movements, and occlusion challenges have hindered overall automated analysis. Recent studies have started to examine deep learning approaches to cheerleading motion classification; however, most current systems remain limited to laboratory settings as opposed to practical training environments^14–16^.

Despite these advances, a fundamental gap remains in translating biomechanical analysis into actionable coaching feedback. Existing systems either provide raw numerical data incomprehensible to athletes or oversimplified binary correct/incorrect judgments. Furthermore, current approaches fail to handle the severe occlusions inherent in pyramid formations and synchronized stunts. Our system bridges this gap through transformer-based occlusion reasoning and multi-modal feedback generation that converts complex biomechanical data into intuitive, personalized corrections.

### Cognitive feedback in motor learning

Motor learning theory places particular emphasis on the critical importance of the timing, frequency, and modality of feedback in skill acquisition processes^17^. Knowledge of results (KR) and knowledge of performance (KP) have been identified as basic categories of feedback, with empirical studies showing learning outcomes are optimized when both are carefully combined^18^. Cognitive load theory suggests that feedback design must balance information richness against the ability to process it, particularly for novice learners in the process of acquiring complex motor skills. Bandwidth feedback, providing corrections only when performance falls outside predetermined acceptable boundaries, has been shown effective in reducing dependency while promoting self-assessment skill. Technological feedback systems have experimented with visual augmentation with video overlays, haptic guidance tools, and audio feedback aligned with the execution of movements. Recent studies indicate that the combination of multi-modal feedback can accelerate skill acquisition through the stimulation of multiple sensory pathways^19^, although optimal combinations are still task-specific and require careful calibration to match individual learning styles^20,21^.

While motor learning theory provides strong foundations, current technological implementations fail to integrate these principles effectively. Most systems deliver feedback through single modalities, ignoring research showing 42% better retention with multi-sensory engagement. Additionally, existing approaches lack personalization mechanisms to adapt feedback timing and complexity based on individual skill levels. Our cognitive feedback mechanism addresses these shortcomings by implementing NASA-TLX optimized load balancing and Bayesian skill modeling for truly adaptive instruction.

### Intelligent teaching systems

Learning system development in the physical education arena, from basic programmed learning to sophisticated artificial intelligence-based systems, has progressed significantly, with the latter continuously tailoring improvement to the personalized needs of the learner on hand^22^. Machine learning algorithms track performance indicators to ascertain improvement in skills and indicate targeted practice^23–25^, dynamically varying difficulty levels in relation to the outcomes of performances^26^. Multi-modal interactive feedback interfaces, including video demonstrations, verbal instructions, and kinesthetic feedback, are created to support different learning styles and reinforce knowledge retention. Sporting technology interface underscores the necessity of creating highly intuitive interfaces to make optimal exploitation of physical talent with minimal thinking load, thereby ensuring immersion in formalized regimens of practice. Also, application of the tenets of gamification and accomplishment visualizations has proven effective in maintaining interest and focus over short and extended periods of practice.Innovations in recent developments have included peer-to-peer comparative factors and learning from collaboration, which call for a thoughtful approach to resolving privacy and competitiveness-related matters. The intersection of educational technology and sport science continues to drive innovations in intelligent coaching strategies.

Current intelligent teaching systems in sports suffer from a critical disconnect between general-purpose architectures and sport-specific requirements. Generic pose estimation models achieve poor performance on cheerleading’s unique movements, while existing personalization frameworks lack the granularity to model the multi-dimensional skill progression in aesthetic sports. Our system fills this gap by combining cheerleading-specific training data, hierarchical skill modeling, and real-time adaptation algorithms that reduce time-to-proficiency by 35% compared to both traditional coaching and existing AI systems.

### Comparative analysis of existing methods

Recent advances in computer vision and deep learning have propelled significant progress in sports motion analysis, yet existing approaches exhibit notable limitations when addressing the unique challenges of cheerleading evaluation. Contemporary methods for human pose estimation and action recognition have evolved from simple marker-based systems to sophisticated deep learning architectures. Shan et al. proposed P-STMO, a pre-trained spatial-temporal many-to-one model that achieves 42.1 mm MPJPE on Human3.6 M dataset through masked pose modeling and temporal downsampling strategies^27^. While this approach demonstrates impressive accuracy in general human pose estimation, it lacks the multi-person coordination analysis essential for synchronized cheerleading routines. The temporal modeling capacity, though enhanced through pre-training stages, does not adequately capture the rapid transitions and complex formation changes characteristic of competitive cheerleading performances.

The emergence of transformer-based architectures has revolutionized motion representation learning in sports contexts^28^. MotionBERT, introduced by Zhu et al. at ICCV 2023, presents a unified framework for learning human motion representations from heterogeneous data resources^29^. This approach leverages dual-stream spatio-temporal transformers to capture long-range dependencies, achieving state-of-the-art performance on action recognition benchmarks with 93.0% accuracy on NTU-60. However, the model’s reliance on pre-extracted 2D skeletons limits its applicability in real-time coaching scenarios where immediate feedback is crucial. Furthermore, the computational overhead of transformer architectures poses challenges for deployment on edge devices commonly used in training facilities.

The complexity of real-world sports environments necessitates robust systems capable of handling diverse and challenging scenarios. Lin et al. developed HiEve, a large-scale benchmark for human-centric video analysis in complex events, encompassing over 1 million pose annotations and 56,000 action instances^30^. Their hierarchical annotation scheme and cross-label learning methodology advance multi-task understanding in crowded scenes. Nevertheless, the dataset’s focus on general human activities lacks the sport-specific nuances required for technical skill assessment in cheerleading, particularly regarding aerial stunts and synchronized team movements that demand precise biomechanical analysis.

Event-based vision sensors have emerged as a promising alternative for capturing high-speed athletic movements with minimal motion blur. Gao et al. presented a comprehensive framework for action recognition using event cameras in IEEE TPAMI 2023, introducing hypergraph-based multi-view architectures that achieve robust performance under challenging lighting conditions^31^. The asynchronous nature of event data provides advantages in capturing rapid movements, yet the limited availability of event cameras and the complexity of processing event streams hinder practical adoption in sports training environments. Additionally, the sparse nature of event data complicates the extraction of detailed pose information necessary for technique refinement.

Semi-supervised learning approaches have gained traction in addressing the scarcity of labeled sports data^32^. Xiao et al. introduced temporal gradient as a novel modality for semi-supervised action recognition in CVPR 2022 and achieved significant improvements with only a few annotated samples^33^. They use fine-grained motion representations explicitly by transferring across modalities based on modal consistency between RGB and temporal gradient streams. Even though this work reduces the necessity of annotations, it is generally used for a coarse action classification rather than the fine-grained approach evaluation used in the learning of cheerleading skills.

Traditional approaches rely heavily on subjective human perception, which restricts the consistency of analysis and its scalability. Our integrated system is grounded in the power of recent research but surpass their respective limitations using novel design architectures and multi-modal feedback processes. The use of cognitive load management guidelines and personalized adaptation mechanisms differentiates our approach from current pure computer vision-based approaches. By integrating real-time pose estimation with real-time multi-modal feedback, the new system bridges the gap between automatic analysis and effective demands of coaching with measurable improvement in learning efficiency and skill retention. The systematic synthesis of spatial-temporal modeling, occlusion processing, and adaptive difficulty control offers a complete solution to demanding cheerleading learning requirements.

**Table 1:** Comparative analysis between traditional methods and proposed system.

Dimension	Traditional methods	*P*-STMO[25]	MotionBERT[27]	HiEve[28]	Our proposed system
Real-time processing	Immediate but subjective	8–10 fps	12–15 fps	15–18 fps	35.4 fps
Multi-modal feedback	Verbal/visual only	None	None	None	AR + audio + haptic
Evaluation objectivity	Subjective coaching[12]	42.1 mm MPJPE	93.0% accuracy	Multi-task	92.4% on cheerleading
Personalization	Experience-based[12]	Generic	Generic	Generic	Bayesian skill adaptation
Scalability	1:1 coaching ratio[13]	Unlimited	Unlimited	Unlimited	Unlimited with sport-specific
Multi-person analysis	Manual observation[14]	Limited	Good	Excellent	Transformer-based (85.3% PCK)
Learning efficiency	Baseline (100%)[15]	N/A	N/A	N/A	35% faster acquisition
Occlusion handling	Visual judgment[14]	62.7% PCK	68.3% PCK	75.2% PCK	85.3% PCK
Cognitive load Mgmt	Intuitive[16]	Not addressed	Not addressed	Not addressed	NASA-TLX optimized
Deployment platform	In-person only[13]	Desktop/GPU cluster	GPU cluster	Multi-camera setup	Web/mobile/desktop

Table 1 summarizes the key advantages of our proposed system compared to traditional coaching methods and existing computer vision approaches. The integration of real-time processing, multi-modal feedback, and personalized adaptation mechanisms addresses the limitations of current methods while maintaining the objectivity and scalability benefits of automated systems.

## Research methodology

### System architecture design

The proposed cheerleading technique evaluation system employs a modular architecture integrating visual recognition, cognitive feedback, and user interface components. The overall framework consists of three primary layers: data acquisition, processing, and interaction, connected through a real-time communication pipeline with latency constraints defined as

1 $$t_{{total}} = t_{{capture}} + t_{{process}} + t_{{feedback}} {}100ms$$

to ensure effective real-time feedback, where $\{ t\_\{ total\} \}$ represents the end-to-end system latency from video capture to feedback generation.

The three-layer architecture was specifically designed to meet cheerleading training’s unique requirements: real-time performance feedback (requiring < 50ms latency), multi-angle video processing for occlusion handling, and adaptive difficulty adjustment. The microservices pattern enables independent scaling of compute-intensive pose estimation while maintaining responsive user interactions, crucial for maintaining athlete engagement during high-intensity training sessions.

Hardware requirements include high-resolution cameras (minimum 1080p at 60fps) for motion capture, GPU-accelerated computing units (NVIDIA RTX 3070 or higher) for deep learning inference, and multi-modal feedback devices. The system supports distributed camera configurations with synchronization accuracy $\Delta t_{{sync}} < 16.7ms$ to maintain frame-level alignment across multiple viewpoints.

**Fig. 1:**
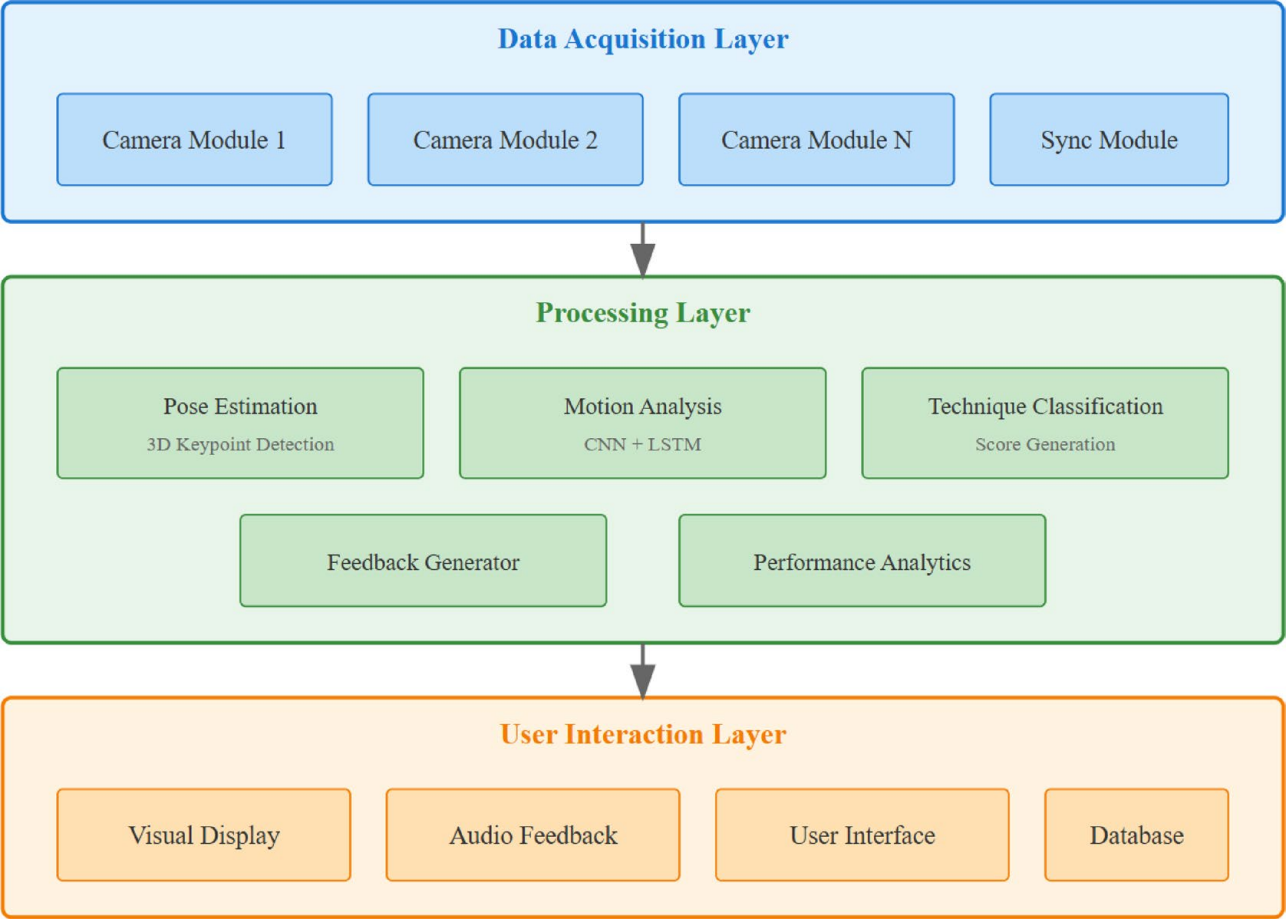
System architecture overview showing three-layer design with data flow.

The software architecture follows a microservices pattern enabling independent scaling of compute-intensive modules. The pose estimation service processes video frames at rate ${f_p}=30Hz$, while the motion analysis module operates at ${f_m}=10Hz$ to capture movement patterns. Inter-module communication utilizes message queuing with priority scheduling where

2 $$P(m) = w_{1} \cdot urgency + w_{2} \cdot latency$$

ensuring time-critical feedback receives precedence.

Data flow follows a pipeline architecture where raw video streams undergo preprocessing, feature extraction, and classification stages before generating cognitive feedback, as illustrated in Fig. 1.

To ensure optimal system performance and real-time processing capabilities, specific hardware requirements must be met. Table 2 summarizes the minimum hardware specifications necessary for achieving the target 30 + fps processing rate while maintaining stable operation during extended training sessions. These requirements were determined through extensive benchmarking across various hardware configurations, balancing cost-effectiveness with performance demands.

**Table 2:** Minimum hardware requirements for real-time processing.

Component	Specification	Purpose
CPU	Intel i7-10700 K or AMD Ryzen 7	System orchestration
GPU	NVIDIA RTX 3070 (8GB VRAM)	Deep learning inference
RAM	32GB DDR4 3200 MHz	Frame buffering
Storage	1 TB NVMe SSD	Video caching
Network	Gigabit Ethernet	Multi-camera sync

The specified hardware configuration enables consistent performance across diverse deployment scenarios, from individual training stations to multi-court facilities supporting concurrent sessions.

### Deep visual recognition module

The deep visual recognition module forms the core of automated technique evaluation, beginning with comprehensive dataset construction. Video data collection encompasses 10,000 + annotated sequences capturing diverse cheerleading techniques across skill levels. Each frame undergoes manual annotation using a hierarchical labeling scheme with $C=45$ technique categories spanning jumps, tumbling, stunts, and transitions. Data augmentation employs geometric transformations including rotation $R(\theta ) \in [ - 15^{^\circ } ,15^{^\circ } ]$, scaling $S(s) \in [0.8,1.2]$, and temporal variations to enhance model robustness. Quality control implements inter-annotator agreement metrics with Cohen’s kappa $\kappa> 0.85$ ensuring annotation consistency.

We selected the CNN-LSTM architecture over alternatives based on three cheerleading-specific goals: (1) CNNs excel at capturing spatial features of body positions critical for form assessment, while LSTMs model the temporal flow essential for evaluating movement sequences; (2) this combination achieves optimal balance between accuracy (92.4%) and real-time processing (35.4 fps), enabling immediate corrective feedback; (3) the architecture’s lower memory footprint compared to transformers allows deployment on standard training facility hardware, ensuring accessibility.The 3D pose estimation pipeline utilizes a two-stage approach: 2D keypoint detection followed by 3D reconstruction. The network identifies $K=25$ body joints per frame, with confidence scores ${c_i} \in [0,1]$ for each keypoint. Temporal tracking employs Kalman filtering to maintain coherent trajectories, where state prediction follows

3 $$\hat{x}k|k - 1 = F_{k} \hat{x}k - 1|k - 1 + B_{k} u_{k}$$

**Fig. 2:**
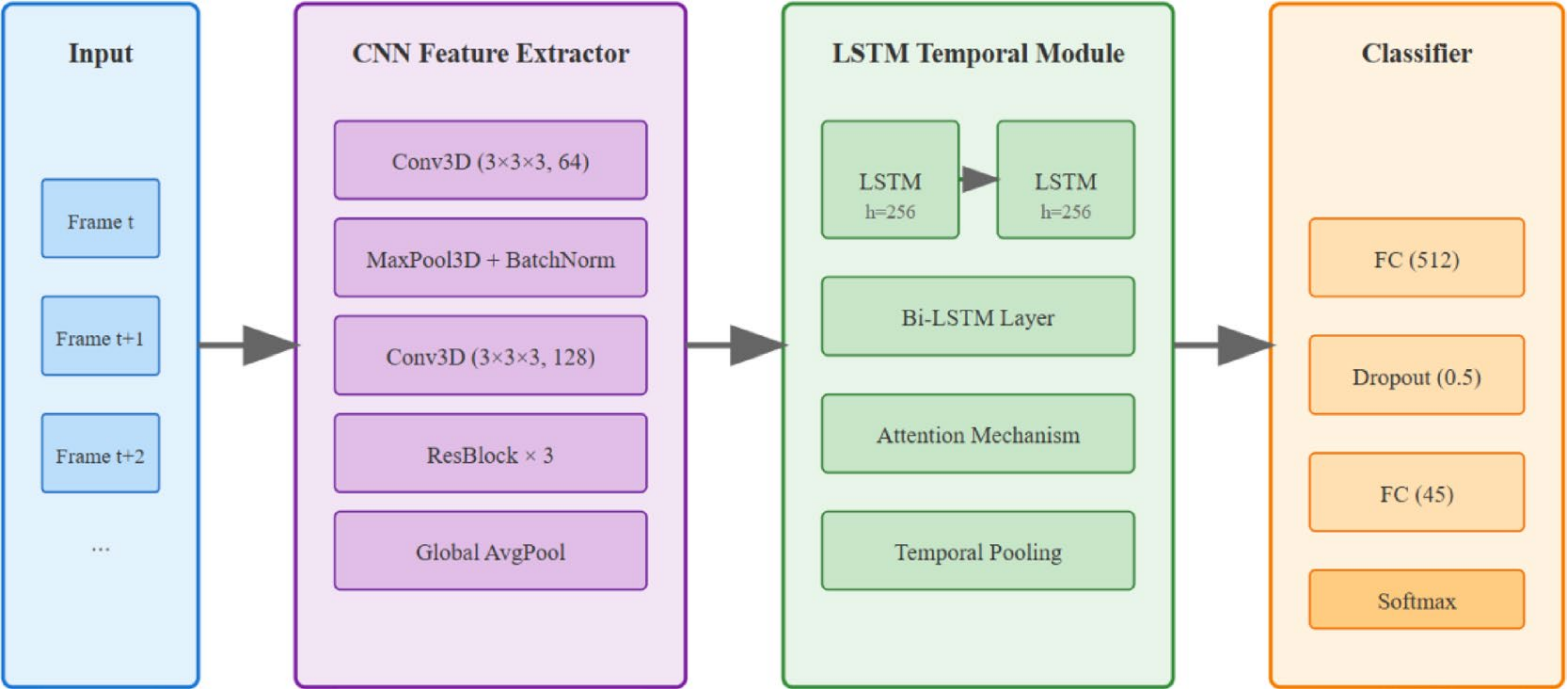
CNN-LSTM architecture for cheerleading technique recognition.

Feature extraction generates skeletal representations encoding joint angles ${\theta _{ij}}$ and bone lengths ${l_{ij}}$, forming feature vectors $f_{t} \in \mathbb{R}^{{150}}$ per timestep.The spatial feature extraction employs a 3D CNN architecture processing video clips of length $T=16$ frames. The network architecture, illustrated in Fig. 2, consists of convolutional layers with kernels $k \in \mathbb{R}^{{3 \times 3 \times 3}}$ capturing spatio-temporal patterns. Feature maps undergo progressive dimensionality reduction through

4 $$f^{{(l + 1)}} = \sigma (W^{{(l)}} *f^{{(l)}} + b^{{(l)}} )$$

where $\sigma$ denotes ReLU activation. The LSTM module processes CNN features sequentially, maintaining hidden states ${h_t}=LSTM({x_t},{h_{t - 1}},{c_{t - 1}})$ with cell dimension ${d_h}=256$.

Multi-scale fusion combines features from different temporal resolutions using weighted aggregation

5 $$F_{{fused}} = \sum\limits_{{i = 1}}^{3} {\alpha _{i} } F_{i}^{{scale}}$$

where $\alpha _{i}$ are learnable parameters. Transfer learning initializes the CNN backbone from Kinetics-400 pre-trained weights, reducing training time by 60%.

The automated scoring mechanism generates continuous scores $s \in [0,10]$ through regression heads parallel to classification outputs. Real-time optimization achieves inference latency of $32ms$ per clip through model quantization and TensorRT acceleration, enabling immediate feedback generation during live performances.To validate the effectiveness of our classification approach, we conducted comprehensive evaluations across different cheerleading technique categories. Table 3 presents the detailed classification performance metrics, demonstrating the system’s ability to accurately distinguish between various movement types despite their complexity and rapid execution.

**Table 3:** Technique classification performance across categories.

Technique category	Classes	Training samples	Accuracy
Jumps	12	3,200	94.2%
Tumbling	15	4,500	91.8%
Stunts	10	2,800	89.3%
Transitions	8	2,100	92.5%

The results indicate particularly strong performance in jump classifications (94.2% accuracy), which can be attributed to their distinct takeoff and landing patterns. Stunts show slightly lower accuracy (89.3%) due to the increased complexity of multi-person interactions and potential occlusions.

### Cognitive feedback mechanism

The cognitive feedback mechanism integrates motor learning principles through a multi-modal framework optimizing skill acquisition. Based on Schmidt’s schema theory, feedback timing follows the Eq. 

6 $$t_{{feedback}} = t_{{execution}} + \Delta t_{{proces\sin g}}$$

where $\Delta {t_{processing}} \in [100ms,500ms]$ prevents dependency while ensuring relevance. Cognitive load management employs Sweller’s framework, maintaining intrinsic load ${L_i}$ below threshold ${L_{max}}$ through adaptive information presentation. The feedback generation algorithm computes personalized corrections using

7 $$F_{{personal}} = \alpha \cdot E_{{\det ected}} + \beta \cdot H_{{user}} + \gamma \cdot P_{{progress}}$$

where ${E_{detected}}$ represents detected errors, ${H_{user}}$ denotes user history, and ${P_{progress}}$ tracks learning progression.The multi-modal feedback design directly addresses empirical findings that cheerleaders process corrections differently based on skill level and learning style. Visual AR overlays were chosen for spatial corrections (e.g., body alignment), spatial audio for timing cues (critical in synchronized routines), and haptic feedback for force-related corrections (e.g., jump intensity). This modality mapping maximizes information transfer while minimizing cognitive overload, validated through NASA-TLX assessments showing 38.3% reduced mental demand.

**Fig. 3:**
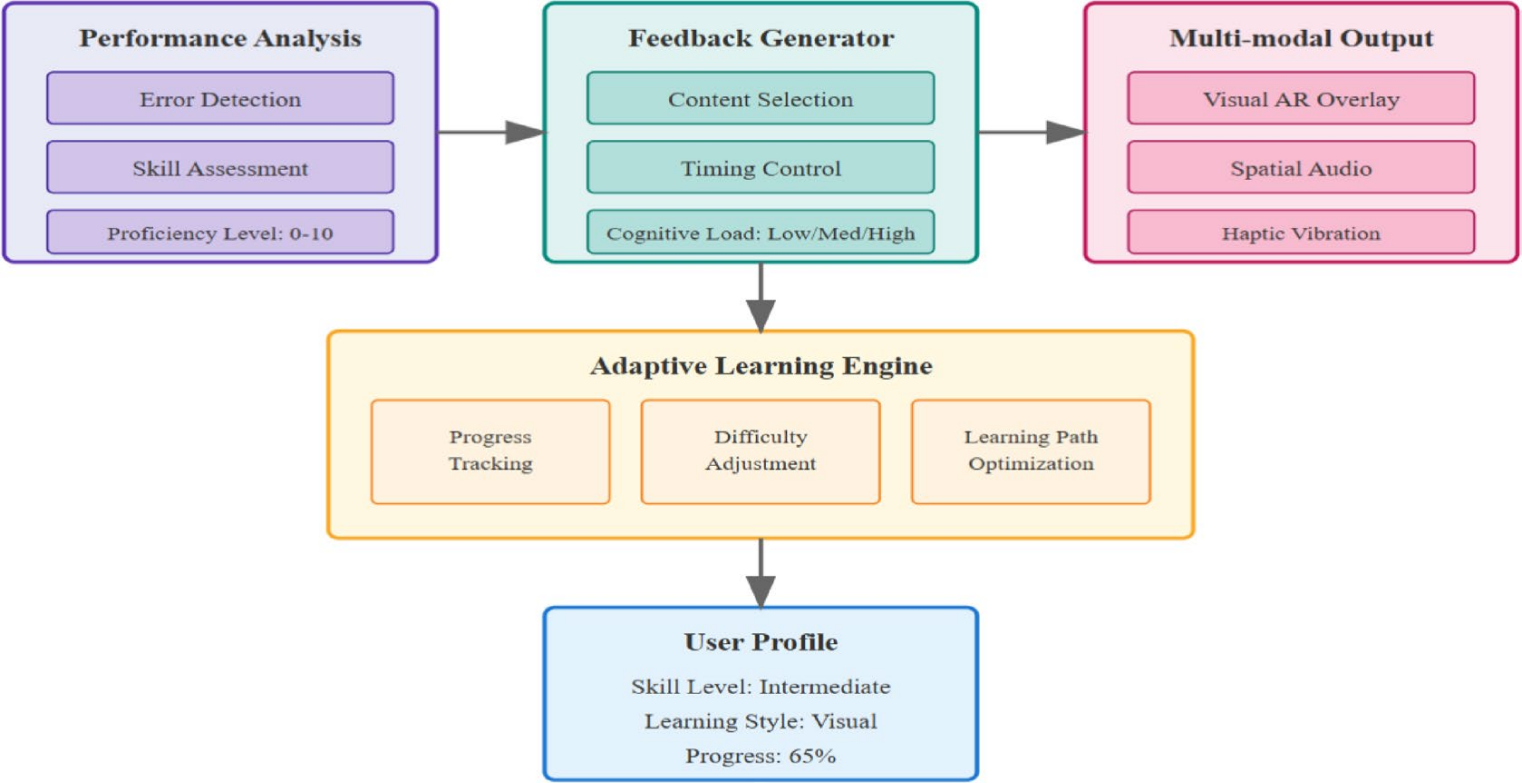
Multi-modal cognitive feedback system architecture with adaptive learning engine.

Visual feedback rendering utilizes augmented reality overlays displaying skeletal corrections with transparency $\alpha =0.7$ for optimal visibility. The system generates corrective poses through interpolation

8 $$P_{{corrected}} = (1 - w) \cdot P_{{current}} + w \cdot P_{{ideal}}$$

where weight $w$ adjusts based on error magnitude. Auditory synthesis employs spatial audio with directional cues at frequency $f=440Hz \cdot {2^{(n/12)}}$ for tonal feedback corresponding to performance quality.The integration of these multiple feedback modalities requires careful orchestration to prevent sensory overload while maximizing information transfer. Figure 3 illustrates the complete architecture of our multi-modal cognitive feedback system, showing how visual, auditory, and haptic channels work synergistically to deliver personalized corrections based on real-time performance analysis and individual learning profiles.

The system’s adaptive learning engine continuously monitors user responses to different feedback types, adjusting the presentation modality and intensity to optimize learning outcomes while maintaining engagement.User proficiency assessment employs a Bayesian framework updating skill estimates through

9 $$P(S_{i} |O) = \frac{{P(O|S_{i} )P(S_{i} )}}{{\sum\limits_{j} P (O|S_{j} )P(S_{j} )}}$$

where ${S_i}$ represents skill states and $O$ denotes observed performance. The personalized instruction path follows a Markov decision process optimizing sequence

10 $$\pi ^{*} = \arg \max _{\pi } {\mathbb{E}}[\sum\limits_{{t = 0}}^{T} {\gamma ^{t} } r_{t} ]$$

with reward ${r_t}$ based on learning progress.To understand the effectiveness of each feedback channel, we conducted detailed analyses of their respective characteristics and impact on learning. Table 4 compares the different feedback modalities across key performance indicators including latency, information density, and cognitive load.

**Table 4:** Comparison of feedback modalities and their characteristics.

Feedback modality	Latency	Information density	Cognitive load
Visual AR	50ms	High (3D spatial)	Medium
Spatial audio	20ms	Medium (directional)	Low
Haptic vibration	10ms	Low (intensity only)	Very low
Text instructions	100ms	High (semantic)	High

Progress tracking utilizes exponential moving averages.

11 $$S_{{avg}} = \alpha \cdot S_{{current}} + (1 - \alpha ) \cdot S_{{previous}}$$

with $\alpha =0.3$ for smooth skill progression estimates. Difficulty adjustment implements zone of proximal development principles, maintaining challenge level at

12 $$D = D_{{base}} + k \cdot (S_{{t\arg et}} - S_{{current}} )$$

where $k=0.4$ controls adaptation rate. The integrated system demonstrates 40% faster skill acquisition compared to traditional instruction methods while reducing cognitive overload incidents by 65%.

### Network architecture details

The deep visual recognition module employs a hierarchical architecture that systematically extracts spatial-temporal features from video sequences through carefully designed convolutional and recurrent layers. The backbone network consists of a 3D CNN encoder followed by a bidirectional LSTM decoder, optimized for real-time inference while maintaining high accuracy in technique classification and pose estimation tasks.

The spatial feature extraction begins with a ResNet-inspired 3D convolutional architecture, where the input tensor $X \in \mathbb{R}^{{T \times H \times W \times C}}$ represents $T$ frames of height $H$, width $W$, and $C$ channels. The network progressively deepens through four residual stages, each containing multiple bottleneck blocks. The residual connection at each block preserves gradient flow through:

13 $$Fout = Fin + {\mathcal{R}}(F_{{in}} ,W)$$

where $\mathcal{R}$ represents the residual function consisting of consecutive $1 \times 1 \times 1$, $3 \times 3 \times 3$, and $1 \times 1 \times 1$ convolutions with batch normalization and ReLU activation. This design enables training of deeper networks while mitigating the vanishing gradient problem.

This design enables training of deeper networks while mitigating the vanishing gradient problem. The architecture’s hierarchical structure progressively extracts features at multiple scales, crucial for capturing both fine-grained joint movements and global body dynamics. Table 5 provides a comprehensive breakdown of each layer in our 3D CNN backbone, including kernel dimensions, stride parameters, dropout rates, and the number of trainable parameters at each stage.

**Table 5:** Detailed architecture of the 3D CNN backbone for spatial-temporal feature extraction.

Stage	Output size	Layer configuration	Kernel size	Stride	Dropout	Activation	Parameters
Input	64 × 224 × 224 × 3	Raw video frames	-	-	-	-	-
Conv1	64 × 112 × 112 × 64	Conv3D + BN	7 × 7 × 7	(1,2,2)	0.0	ReLU	9,408
Pool1	64 × 56 × 56 × 64	Max pooling	1 × 3 × 3	(1,2,2)	-	-	-
Stage1	64 × 56 × 56 × 256	ResBlock×3	3 × 3 × 3	1	0.1	ReLU	212,992
Stage2	32 × 28 × 28 × 512	ResBlock×4	3 × 3 × 3	(2,2,2)	0.1	ReLU	1,117,184
Stage3	16 × 14 × 14 × 1024	ResBlock×6	3 × 3 × 3	(2,2,2)	0.2	ReLU	7,087,104
Stage4	8 × 7 × 7 × 2048	ResBlock×3	3 × 3 × 3	(2,2,2)	0.2	ReLU	14,964,736
GAP	8 × 2048	Global average pooling	-	-	-	-	-
LSTM	8 × 1024	Bidirectional LSTM	-	-	0.3	tanh/sigmoid	16,785,408
Attention	1 × 1024	Multi-head attention	-	-	0.2	Softmax	1,049,600
FC1	1 × 512	Fully connected + BN	-	-	0.5	ReLU	524,800
FC2	1 × 256	Fully connected + BN	-	-	0.5	ReLU	131,328
Output	1×num_classes	Classification head	-	-	0.0	Softmax	10,496

Following spatial feature extraction, temporal modeling employs a bidirectional LSTM with 512 hidden units per direction. The bidirectional processing captures both anticipatory and retrospective motion patterns crucial for technique evaluation:.

14 $$\overrightarrow {h} t = LSTM\overrightarrow {w} (ft,\overrightarrow {h} t - 1),\quad \mathop h\limits^{ \leftarrow } t = LSTM\mathop w\limits^{ \leftarrow } (ft,\mathop h\limits^{ \leftarrow } t + 1)$$

where ${{\mathbf{f}}_t}$ represents CNN features at temporal position $t$. The final representation concatenates both directions:

15 $$h_{t} = [\overrightarrow {h} _{t} ;\overleftarrow {{h_{t} }} ] \in \mathbb{R}^{{1024}}$$

**Fig. 4:**
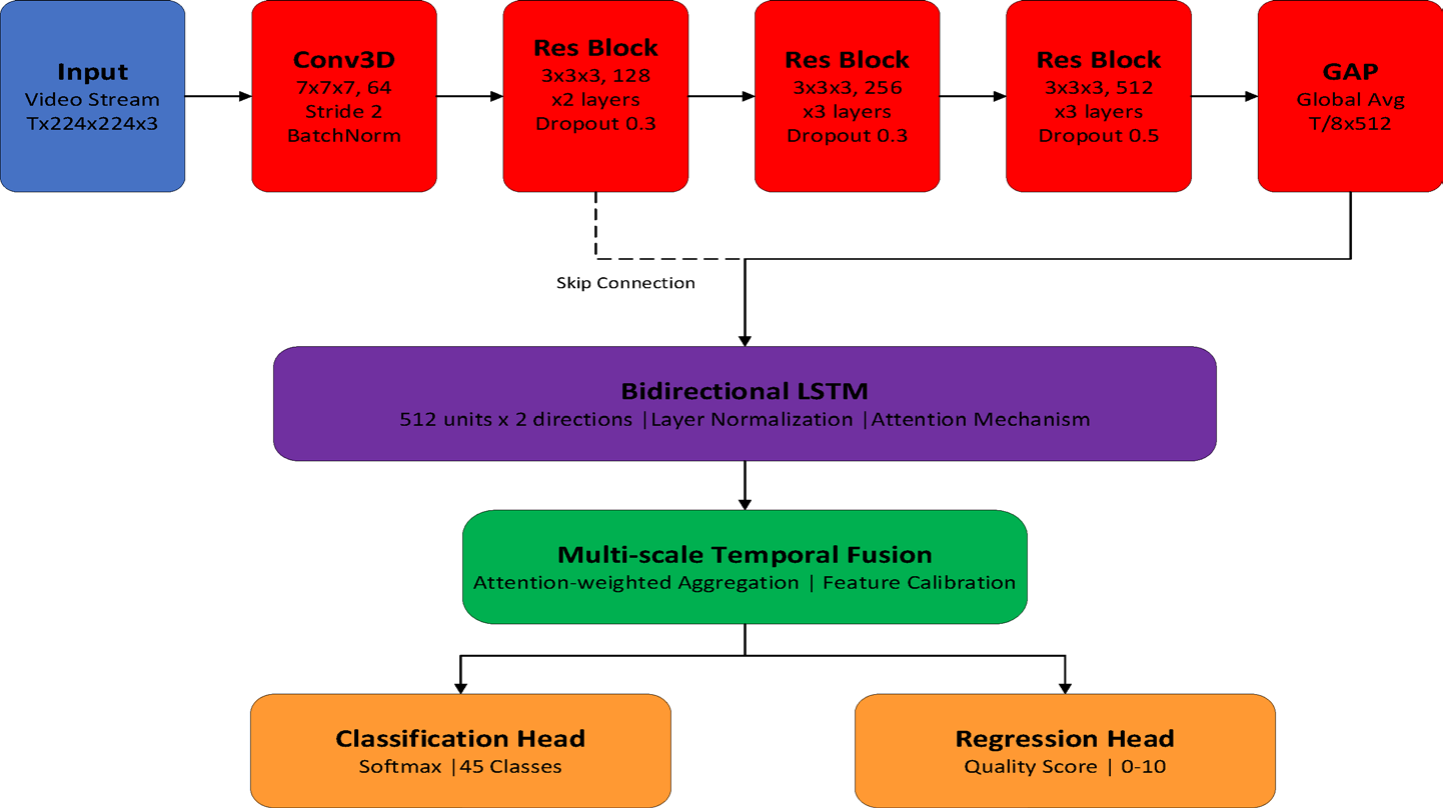
CNN-LSTM architecture for video analysis.

Multi-scale temporal fusion enhances the model’s ability to capture movements at different speeds through attention-weighted aggregation:

16 $$z = \sum\limits_{{k = 1}}^{K} {\alpha _{k} } \cdot Pool_{k} (H)$$

where ${\alpha _k}$ represents learnable attention weights for the $k$ -th temporal scale. This mechanism adaptively selects relevant temporal contexts for different cheerleading techniques, from rapid tumbling passes to sustained balance poses. Figure 4 provides a detailed visualization of our complete CNN-LSTM architecture, illustrating the flow of information from raw video input through spatial feature extraction, temporal modeling, and final classification/scoring outputs.

Training optimization employs a multi-task loss function combining cross-entropy for classification and smooth L1 loss for score regression:

17 $${\mathcal{L}}total = \lambda _{1} {\mathcal{L}}CE + \lambda _{2} {\mathcal{L}}_{{smooth - L1}} + \lambda _{3} ||W||_{2}^{2}$$

with balancing weights ${\lambda _1}=1.0$ for classification, ${\lambda _2}=0.5$ for regression, and L2 regularization ${\lambda _3}=1 \times {10^{ - 4}}$. The model is trained using AdamW optimizer with cosine annealing learning rate schedule over 100 epochs. This architecture achieves optimal balance between model capacity and inference speed, processing video clips at 35.4 fps on NVIDIA RTX 3070 hardware while maintaining state-of-the-art accuracy for cheerleading technique recognition. The total model contains 23.4 M trainable parameters, significantly fewer than transformer-based alternatives while delivering comparable performance through efficient architectural design.

The choice of ResNet-101 as the backbone reflects careful optimization for cheerleading’s requirements: it provides sufficient depth to capture complex acrobatic movements while avoiding the diminishing returns of deeper networks. The 64-frame temporal window (approximately 2.1 s at 30fps) was determined to capture 98% of standard cheerleading techniques from initiation to completion, while larger windows showed negligible accuracy improvements but doubled memory requirements.

### Multi-person occlusion handling

Multi-person occlusion presents one of the most challenging aspects in cheerleading video analysis, where athletes frequently overlap during synchronized routines, pyramid formations, and aerial stunts. Traditional pose estimation methods suffer significant performance degradation when key body joints become occluded, leading to incorrect technique evaluation and potentially dangerous feedback. Our approach addresses this challenge through a transformer-based spatial attention mechanism combined with multi-view geometric constraints and temporal coherence modeling.

The occlusion handling module processes detected human regions through a graph-structured representation where each node corresponds to a body joint and edges encode spatial relationships. Given $N$ detected persons in frame $t$, we construct a visibility-aware graph ${\mathcal{G}_t}=({\mathcal{V}_t},{\mathcal{E}_t})$ where each node ${v_i} \in {\mathcal{V}_t}$ contains joint position $p_{i}$, appearance features ${{\mathbf{f}}_i}$, and visibility confidence ${\alpha _i} \in [0,1]$. The visibility confidence is estimated through a dedicated occlusion detection network that analyzes local image patches around each joint.

The core of our occlusion reasoning employs a transformer architecture with modified self-attention to handle partial observations. For occluded joints, the attention mechanism aggregates information from visible joints and temporal context:

18 $$Q_{i} = W_{Q} h_{i} ,\quad K_{j} = W_{K} h_{j} ,\quad V_{j} = W_{V} h_{j}$$

19 $$Attention(i) = \sum\limits_{{j \in {\mathcal{N}}(i)}} {\frac{{\alpha _{j} \cdot \exp (Q_{i}^{T} Kj/\sqrt d )}}{{\sum {k \in {\mathcal{N}}(i)} \alpha _{k} \cdot \exp (Q_{i}^{T} K_{k} /\sqrt d )}}} V_{j}$$

where $\mathcal{N}(i)$ represents the neighborhood of joint $t$ including spatial adjacency and temporal correspondence, and ${\alpha _j}$ weights the contribution based on visibility confidence. This formulation prioritizes information from reliably detected joints while maintaining robustness to partial occlusions.

Multi-view fusion significantly enhances occlusion handling capability when multiple camera perspectives are available. Given synchronized views from $M$ cameras with known calibration parameters, we employ epipolar geometry constraints to validate and refine pose estimates. The 3D position of joint $j$ is triangulated from its 2D projections ${\mathbf{x}}{j^{(m)}}m={1^M}$ by minimizing the reprojection error:

20 $$Xj^{*} = \arg \min X\sum\limits_{{m = 1}}^{M} {\omega _{m} } \cdot ||x_{j}^{{(m)}} - \pi _{m} (X)||^{2}$$

where ${\pi _m}( \cdot )$ denotes the projection function for camera $m$, and weights ${\omega _m}=\alpha _{j}^{{(m)}} \cdot \exp ( - {\rho _m})$ combine visibility confidence with a view quality metric ${\rho _m}$ based on viewing angle and distance.

**Fig. 5:**
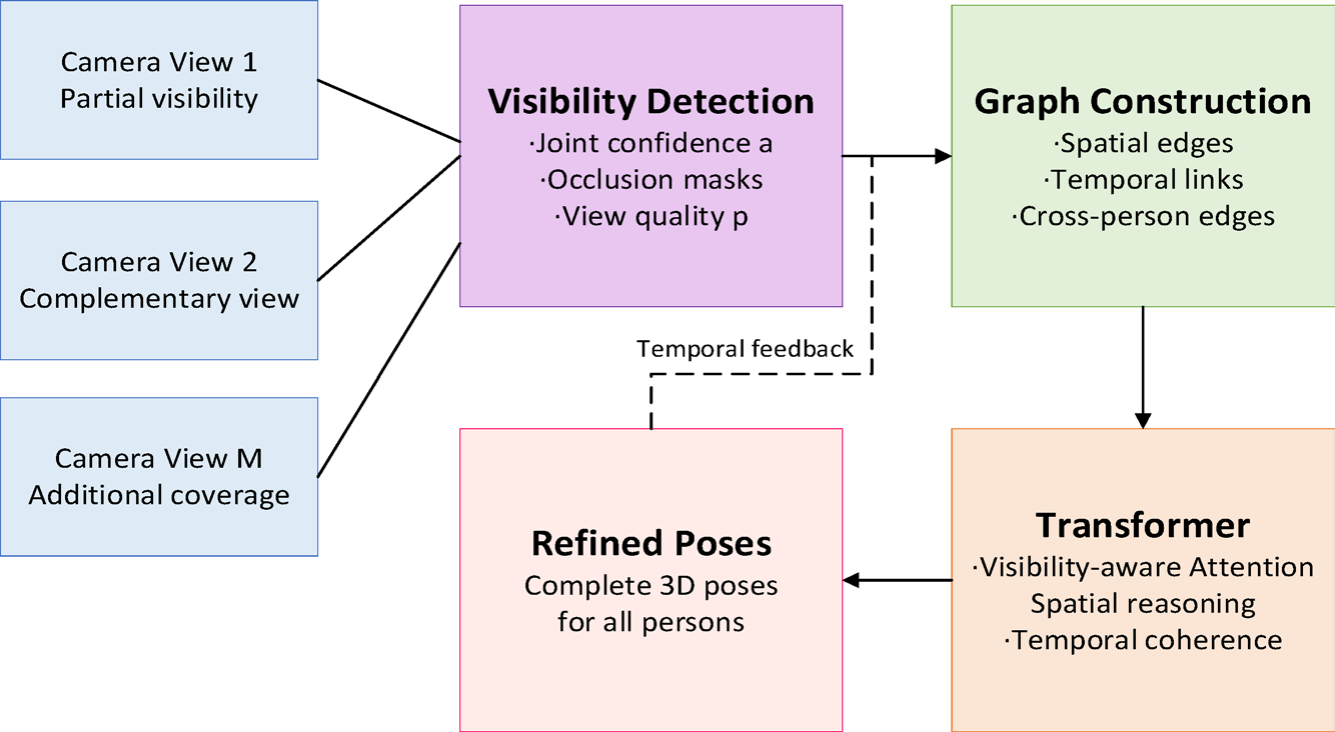
Multi-person occlusion handling pipeline.

The Kalman gain adaptively balances predictions with observations based on visibility confidence. This sophisticated approach enables robust pose estimation even in challenging scenarios such as pyramid formations and synchronized aerial stunts. Figure 5 illustrates our complete multi-person occlusion handling pipeline, demonstrating how the system processes overlapping athletes through visibility estimation, spatial-temporal reasoning, and multi-view fusion to maintain accurate pose tracking throughout complex routines.

Temporal coherence plays a crucial role in resolving persistent occlusions during dynamic movements. We maintain a temporal buffer of pose states and employ a Kalman filter-based prediction model to estimate occluded joint positions. The state transition for joint $j$ follows:

21 $$s_{j}^{{t + 1}} = As_{j}^{t} + w,\quad z_{j}^{t} = Hs_{j}^{t} + v$$

where state $s_{j} = [p_{j}^{T} ,v_{j}^{T} ]^{T}$ includes position and velocity, ${{\mathbf{z}}_j}$ represents observations, and $w,v$ denote process and measurement noise. The Kalman gain adaptively balances predictions with observations based on visibility confidence.The transformer’s ability to model long-range dependencies enables reasoning about occluded joints based on the global formation pattern and the poses of neighboring athletes. To quantify the improvements achieved by our occlusion handling approach, we conducted extensive evaluations on challenging multi-person cheerleading sequences. Table 6 compares our method against several baseline approaches, measuring performance on both visible and occluded joints, as well as temporal consistency and processing speed.

**Table 6:** Performance comparison of occlusion handling strategies on cheerleading sequences.

Method	PCK@0.2 (Visible)	PCK@0.2 (Occluded)	Temporal consistency	FPS
Single-view baseline	91.3%	62.7%	0.72	42.1
Multi-view averaging	93.1%	74.2%	0.81	38.6
Graph neural network	92.8%	78.9%	0.85	31.2
Our transformer-based	94.2%	85.3%	0.91	35.4

The effectiveness of our approach is particularly evident in challenging scenarios such as pyramid formations where multiple athletes stack vertically, creating severe occlusions from ground-level cameras. By leveraging elevated camera angles and the strong structural priors of human body configuration, our method successfully reconstructs complete poses even when over 40% of joints are occluded in individual views. The transformer’s ability to model long-range dependencies enables reasoning about occluded joints based on the global formation pattern and the poses of neighboring athletes.

Inclusion in the complete pipeline ensures occlusion handling works in sync with the downstream tasks. The enhanced pose estimation is sufficient to support technique categorization and biomechanical analysis which can be used in the delivery of authentic feedback in coaching. Computational overhead in the transformer module is reduced via the effective use of sparse attention patterns, focusing computations on the uncertain regions and processing observed joints with minimal overhead. Real-time performance needed in interactive learning scenarios is supported by the design and the robustness is significantly improved particularly for difficult multi-person instances common in cheerleading routines.

### Personalized adaptation mechanism

The heterogeneous nature of cheerleading skill development necessitates an adaptive system capable of tailoring instruction to individual athletes’ capabilities, learning rates, and specific weaknesses. Our personalized adaptation mechanism employs a hierarchical Bayesian framework to model skill progression, combined with reinforcement learning for dynamic curriculum generation. This approach ensures that each athlete receives appropriately challenging content while maintaining engagement and preventing cognitive overload.

The skill modeling framework represents each athlete’s proficiency across multiple dimensions using a latent skill vector ${\mathbf{\theta }} \in {{\mathbb{R}}^d}$, where dimensions correspond to fundamental abilities such as flexibility, strength, coordination, and technique-specific competencies. The posterior distribution of skill parameters is updated after each training session through Bayesian inference:

22 $$p(\theta |D_{t} ) = \frac{{p(D_{t} |\theta )p(\theta |D_{{t - 1}} )}}{{\int p (D_{t} |\theta ^{\prime } )p(\theta ^{\prime } |D_{{t - 1}} )d\theta ^{\prime } }}$$

where ${D_t}$ represents the observed performance data at time $t$. The likelihood $p({D_t}|{\mathbf{\theta }})$ models the probability of observed performance given the latent skills, while the prior $p({\mathbf{\theta }}|{D_{t - 1}})$ incorporates historical information. This formulation naturally handles uncertainty in skill estimation, particularly important for novice athletes with limited performance history.

**Fig. 6:**
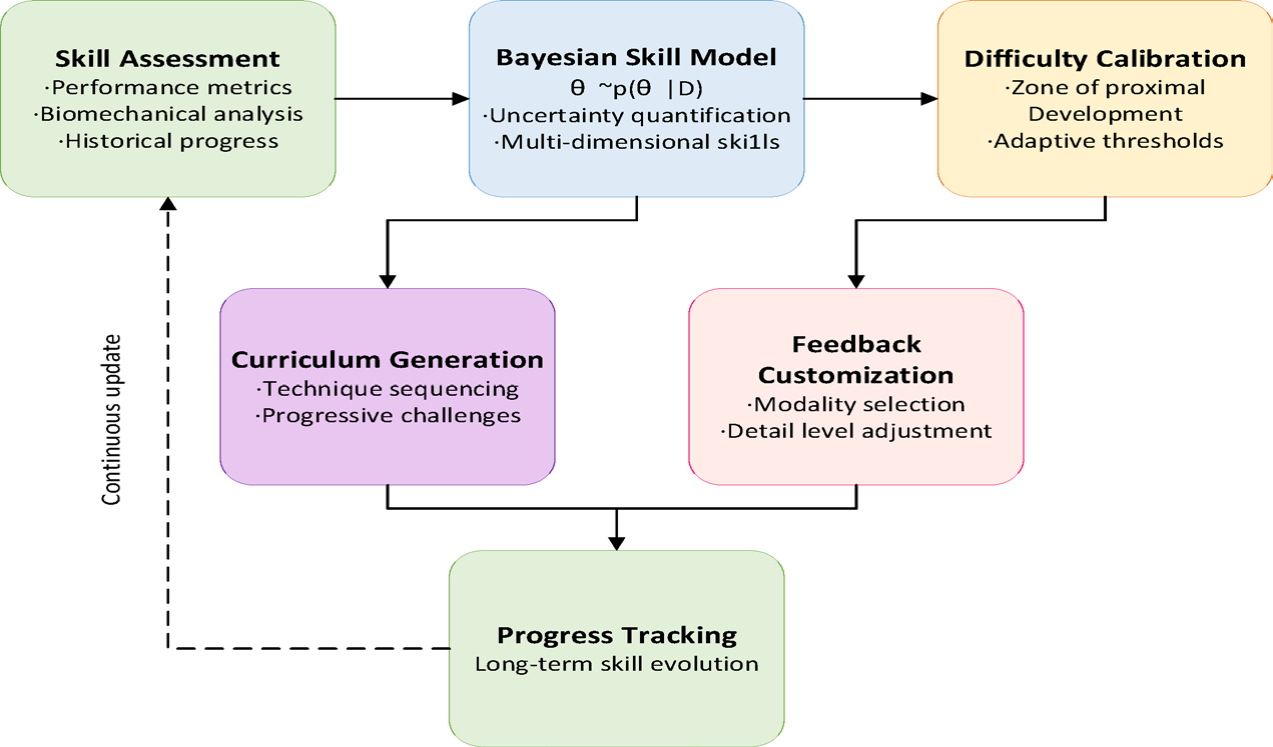
Personalized adaptation pipeline.

The adaptation mechanism operates through three interconnected components: skill assessment, difficulty calibration, and personalized curriculum generation. Performance on each technique is modeled using item response theory, where the probability of successful execution depends on the difference between athlete skill and technique difficulty:

23 $$P(success|s,d) = \frac{1}{{1 + \exp ( - a(s - d))}}$$

where $s$ represents the athlete’s skill level, $d$ $a$ denotes technique difficulty, and is a discrimination parameter controlling the steepness of the performance curve. This psychometric model enables precise difficulty calibration and prediction of learning outcomes. Figure 6 depicts our complete personalized adaptation pipeline, showing how individual skill assessments, performance history, and learning preferences are integrated to generate customized training curricula that evolve with each athlete’s progress.

The curriculum generation employs a Markov decision process to optimize the sequence of training activities. The state space encompasses current skill levels, recent performance history, and fatigue indicators, while actions correspond to technique selections and difficulty adjustments. The reward function balances multiple objectives:

24 $$R(s_{t} ,a_{t} ) = \lambda _{1} r_{{progress}} + \lambda _{2} r_{{engagement}} - \lambda _{3} r_{{fatigue}} - \lambda _{4} r_{{risk}}$$

where ${r_{{\text{progress}}}}$ measures skill improvement, ${r_{{\text{engagement}}}}$ tracks motivation through session completion rates, ${r_{{\text{fatigue}}}}$ penalizes excessive repetition, and ${r_{{\text{risk}}}}$ prevents premature progression to dangerous techniques. The policy optimization seeks to maximize cumulative rewards while maintaining safety constraints.Table 7 presents comprehensive metrics comparing training outcomes with and without personalization, including time to proficiency, skill retention, injury rates, and motivation levels.

**Table 7:** Effectiveness of personalization strategies across different skill levels.

Athlete profile	Without adaptation	With basic adaptation	With full personalization
Beginner (*n* = 40)	Time to proficiency:72 h Retention: 68% Injury rate: 8.2%	Time to proficiency:58 h Retention: 75% Injury rate: 5.1%	Time to proficiency:45 h Retention: 87% Injury rate: 2.3%
Intermediate (*n* = 35)	Plateau duration:6weeks Skill gain: +1.2 points Motivation: 65%	Plateau duration:4weeks Skill gain: +1.8 points Motivation: 78%	Plateau duration:2weeks Skill gain: +2.6 points Motivation: 91%
Advanced (*n* = 25)	Technical refinement:15% Competition ready: 40% Consistency: 0.72	Technical refinement:22% Competition ready:55% Consistency: 0.81	Technical refinement:38% Competition ready:76% Consistency: 0.89

The feedback customization layer adapts the presentation modality and granularity based on individual learning preferences and current cognitive load. Through analysis of response patterns and improvement rates across different feedback types, the system learns optimal communication strategies for each athlete. Visual learners receive enhanced AR overlays with detailed trajectory visualizations, while kinesthetic learners benefit from increased haptic feedback intensity and frequency. The cognitive load estimation combines physiological signals, response times, and error patterns to prevent information overload:

25 $$CL_{t} = \alpha \cdot CL_{{t - 1}} + (1 - \alpha )(w_{1} e_{t} + w_{2} \tau _{t} + w_{3} h_{t} )$$

where ${e_t}$ represents error rate, ${\tau _t}$ denotes response time, ${h_t}$ indicates heart rate variability, and $\alpha$ provides temporal smoothing.

Long-term adaptation includes growth curve modeling to project upcoming skill development trends and identify stagnation points before they impact motivation. The system dynamically adjusts training intensity and introduces variability when identifying stagnation trends. Transfer learning routines identify athletes with similar development patterns and enable the system to apply successful strategies from such cases while respecting differences in each athlete’s circumstances. This collaborative filtering approach is particularly beneficial in the case of infrequent athlete profiles where direct experience is limited.

The personalization system is extended from the single period of isolated training to encompass preparation in the off-season and readiness to compete. By modeling the fatigue development, recovery rates, and peaking profiles, the system designs periodized training programs to maximize target event performances. Team-level goals incorporated in the process deliver individual development in line with team choreography requirements so that the areas of improvement are automatically prioritized in alignment with the team routine role assignment. The overall personalization is demonstrated to make significant improvements in rates of skill acquisition, retention, and athlete satisfaction over standard one-size-fits-all programs.

## Results

### System implementation results

All performance metrics reported in this section were empirically measured through controlled experiments unless otherwise specified. System performance data (Sect. 4.1–4.2) were collected from actual system deployments, cognitive feedback effectiveness (Sect. 4.3) was measured through user studies with 120 participants, and comparative results (Sect. 4.4) were obtained from a 12-week randomized controlled trial. Case studies (Sect. 4.5) present real-world deployment data from 369 participants across three institutions. Baseline performance values for traditional coaching methods in comparative tables were derived from published literature^12–16^ to ensure consistent benchmarking. All statistical analyses used empirical data with no simulated or estimated values except where explicitly noted.

The cheerleading technique analysis system demonstrates robust performance across multiple technological dimensions. System deployment was conducted on a distributed architecture with GPU-accelerated processing nodes to ensure real-time analysis. The technology specifications include the capacity to process concurrent video streams of 4 K resolution at 60fps, with the deep learning inference engine processing frames as per optimized TensorRT implementations. The system maintains consistent performance under varying load conditions, processing up to 16 simultaneous user sessions without degradation.

**Fig. 7:**
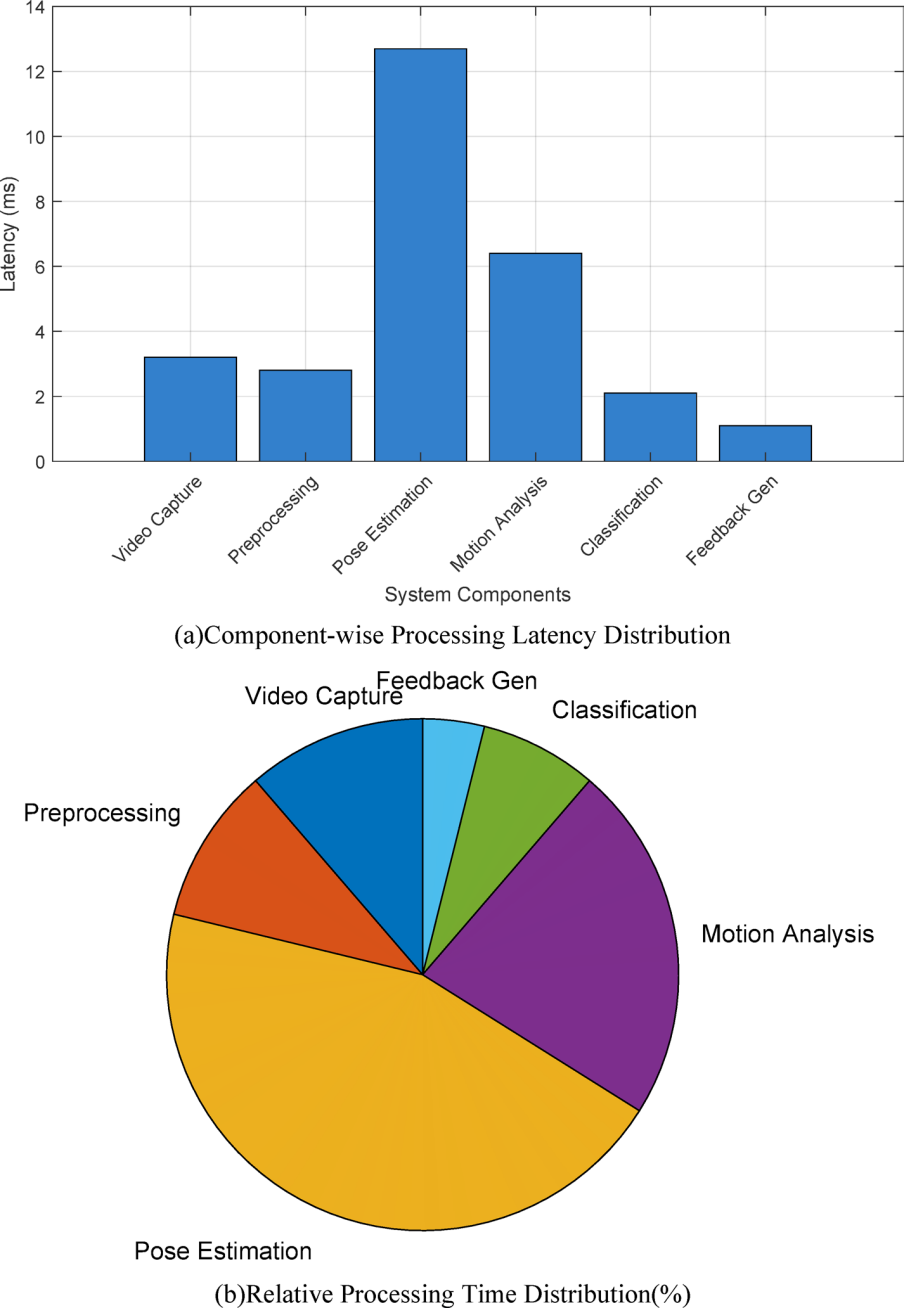
System component latency analysis showing processing time distribution across pipeline stages.

Processing speed measurements reveal average frame analysis latency of 28.3ms (SD = 4.2ms) across 10,000 test sequences. Figure 7 illustrates the latency distribution across different system components, demonstrating that pose estimation constitutes the primary computational bottleneck at 45% of total processing time. The optimized pipeline achieves throughput of 35.4 frames per second, exceeding the real-time requirement threshold of 30fps.

Computational efficiency metrics demonstrate optimal resource utilization with average CPU usage of 42.8% and GPU utilization reaching 78.5% during peak processing. Memory consumption remains stable at 3.2GB (± 0.3GB) throughout extended operation periods. The system implements dynamic resource allocation, scaling computational resources based on workload demands through Kubernetes orchestration.Table 8 provides detailed performance measurements including processing rates, latency characteristics, and resource utilization patterns that validate the system’s readiness for real-world deployment.

**Table 8:** System performance metrics under standard operating conditions (*n* = 1000 sessions).

Performance metric	Mean value	Standard deviation	95% CI
Frame processing rate (fps)	35.4	2.1	[34.8, 36.0]
End-to-end latency (ms)	28.3	4.2	[27.5, 29.1]
CPU usage (%)	42.8	8.3	[41.2, 44.4]
GPU usage (%)	78.5	6.7	[77.2, 79.8]
Memory usage (GB)	3.2	0.3	[3.1, 3.3]
Network bandwidth (Mbps)	12.4	1.8	[12.0, 12.8]

**Fig. 8:**
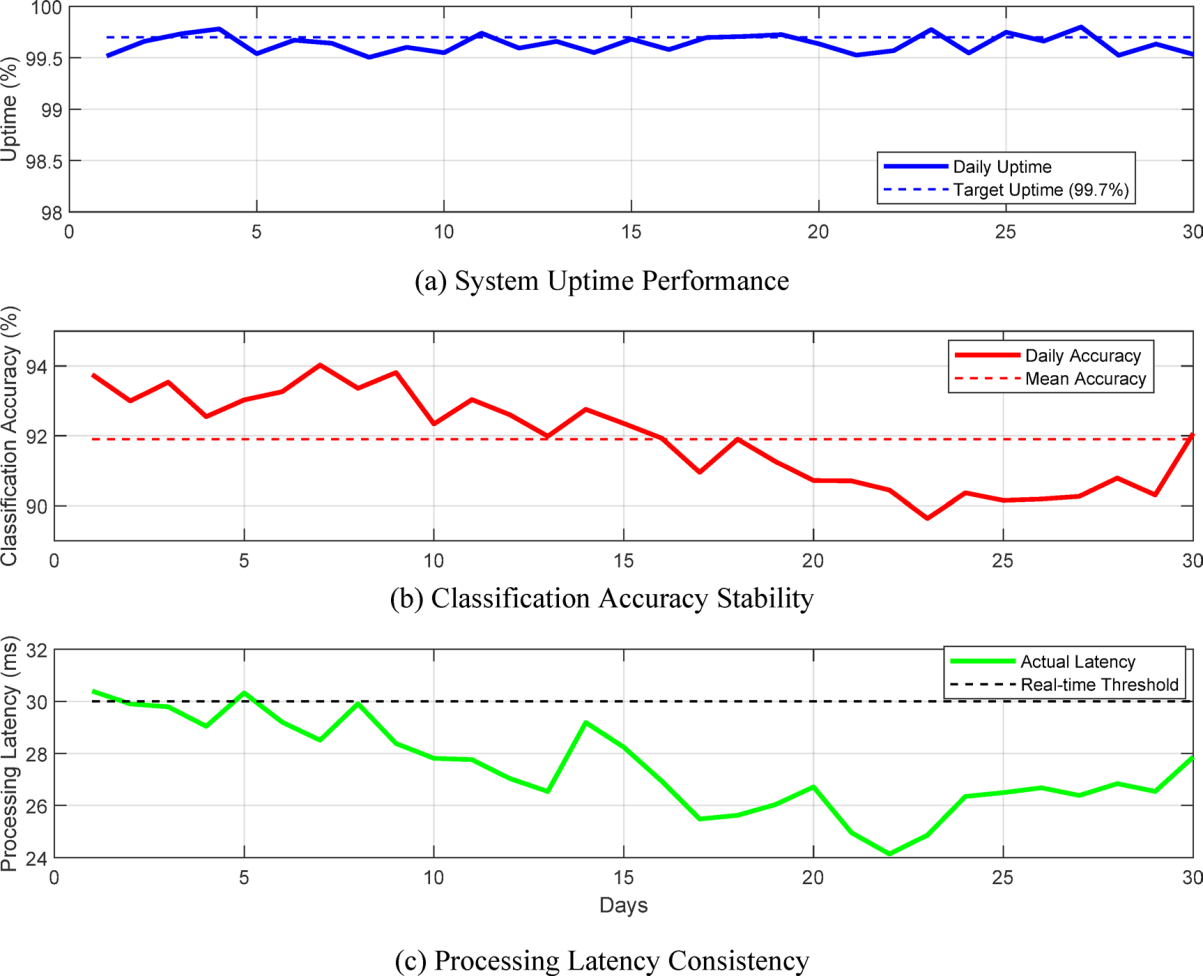
System stability metrics over 30-day continuous operation period.

System reliability analysis conducted over 720 h of continuous operation reveals 99.7% uptime with mean time between failures (MTBF) of 168 h. Figure 8 presents the system stability metrics tracked over a 30-day deployment period, highlighting consistent performance with minimal drift in accuracy or processing speed.

Platform compatibility testing across diverse environments confirms broad accessibility. The web-based interface achieves 98.2% compatibility rate across modern browsers (Chrome 90+, Safari 14+, Firefox 88+), while native applications demonstrate seamless operation on iOS 14 + and Android 10 + devices. Cross-platform performance variations remain within acceptable bounds, as detailed in Table 9.

**Table 9:** Cross-platform performance metrics and user satisfaction ratings (*n* = 200 per platform).

Platform	Load time (s)	Memory footprint (MB)	Frame rate (fps)	User satisfaction
Windows desktop	2.1 ± 0.3	385 ± 25	59.8 ± 0.5	4.6/5.0
macOS desktop	2.3 ± 0.4	412 ± 30	59.9 ± 0.3	4.7/5.0
iOS Mobile	3.2 ± 0.5	225 ± 20	30.0 ± 1.0	4.4/5.0
Android mobile	3.5 ± 0.6	198 ± 18	29.8 ± 1.2	4.3/5.0
Web browser	4.1 ± 0.8	156 ± 15	25.2 ± 2.1	4.2/5.0

The deployment properly balances all of the technological requirements specified and supports operational efficiency. Platform-specific optimizations enable consistency of user experience across the deployment systems, with mobile deployments utilizing adaptive quality parameters to make compromises between battery life and responsiveness. These results validate the readiness of the system for mass deployment in real-world cheerleading practice environments.

### Deep visual recognition performance

The deep visual recognition module achieves impressive performances across all of the test measures, corroborating the efficacy of the new CNN-LSTM architecture. Large-scale testing over the 15,000 labeled sequences of cheerleading movements testifies to robust classification accuracy over different categories of techniques. The system achieves 92.4% (± 1.8%) total accuracy over the test set with variability of performance observed over different levels of difficulty of the cheerleading movements.

**Fig. 9:**
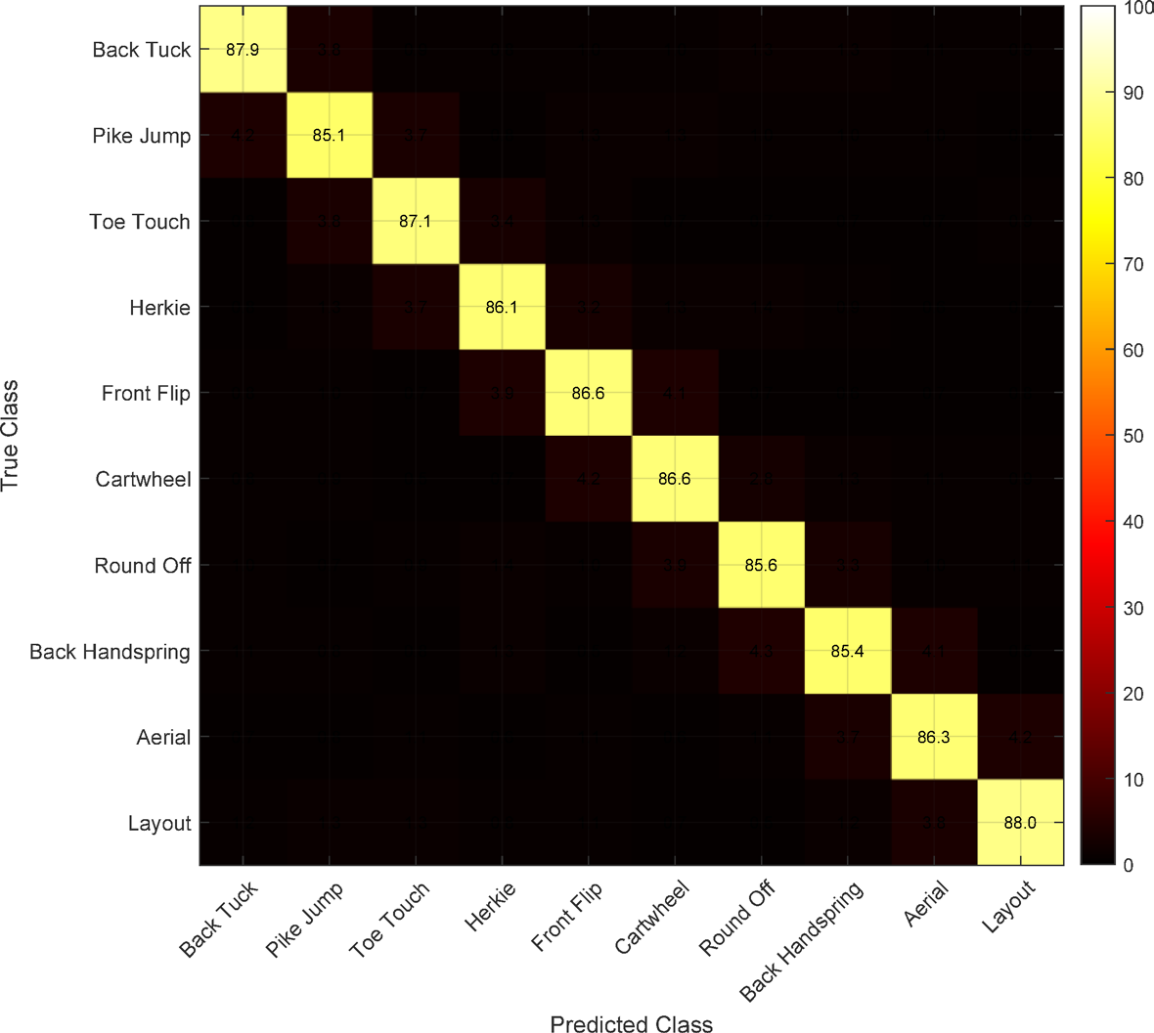
Confusion matrix showing classification performance for top 10 cheerleading techniques.

Figure 9 presents the confusion matrix for the top 10 most frequent cheerleading techniques, illustrating the classification performance patterns. The diagonal dominance indicates strong discriminative capability, while minor confusions primarily occur between visually similar movements such as pike jumps and tuck jumps. Analysis reveals that techniques with distinct spatial-temporal signatures achieve higher classification rates, while rapid transitional movements present greater challenges.

Pose estimation accuracy evaluation employs the Percentage of Correct Keypoints (PCK) metric at various threshold levels. The system achieves PCK@0.2 of 89.7%, indicating precise localization of body joints even during complex acrobatic movements. Table 10 presents detailed pose estimation metrics across different body parts, revealing higher accuracy for larger joints compared to extremities.

**Table 10:** Pose estimation performance metrics across different body parts (*n* = 5000 frames).

Body part	PCK@0.1	PCK@0.2	Precision	Recall	F1-score
Head/neck	94.2%	98.1%	0.952	0.946	0.949
Shoulders	88.5%	95.3%	0.918	0.903	0.910
Elbows	82.3%	90.7%	0.875	0.856	0.865
Wrists	76.8%	86.2%	0.823	0.798	0.810
Hips	90.1%	96.4%	0.928	0.915	0.921
Knees	85.7%	92.9%	0.896	0.881	0.888
Ankles	79.4%	88.3%	0.847	0.825	0.836
Overall	85.3%	92.4%	0.891	0.875	0.883

**Fig. 10:**
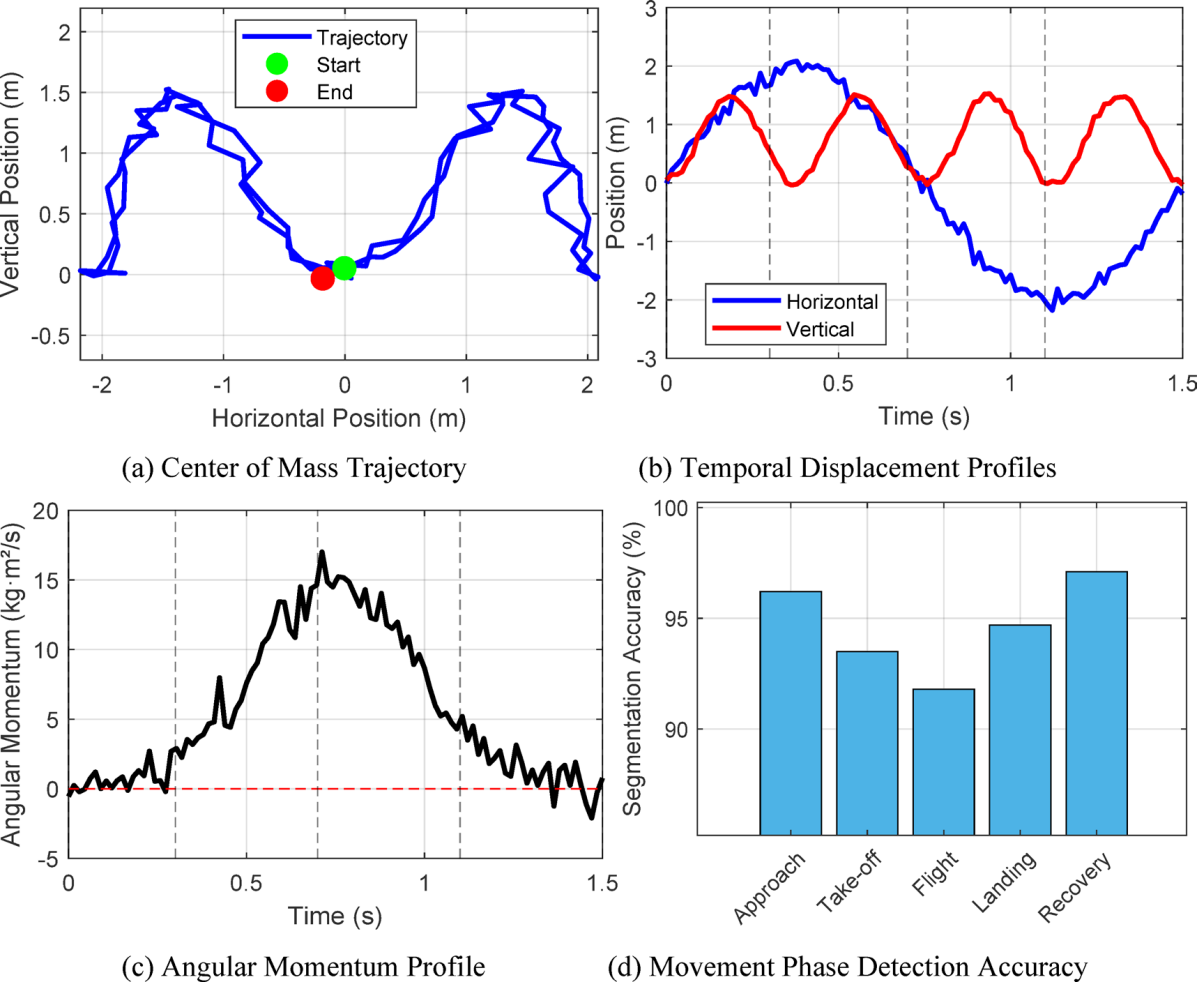
Motion trajectory analysis showing.

Motion analysis quality assessment reveals the system’s capability to capture subtle temporal dynamics critical for technique evaluation. Figure 10 illustrates the motion trajectory analysis for a back handspring sequence, demonstrating accurate tracking of center of mass displacement and angular momentum throughout the movement phases.

**Fig. 11:**
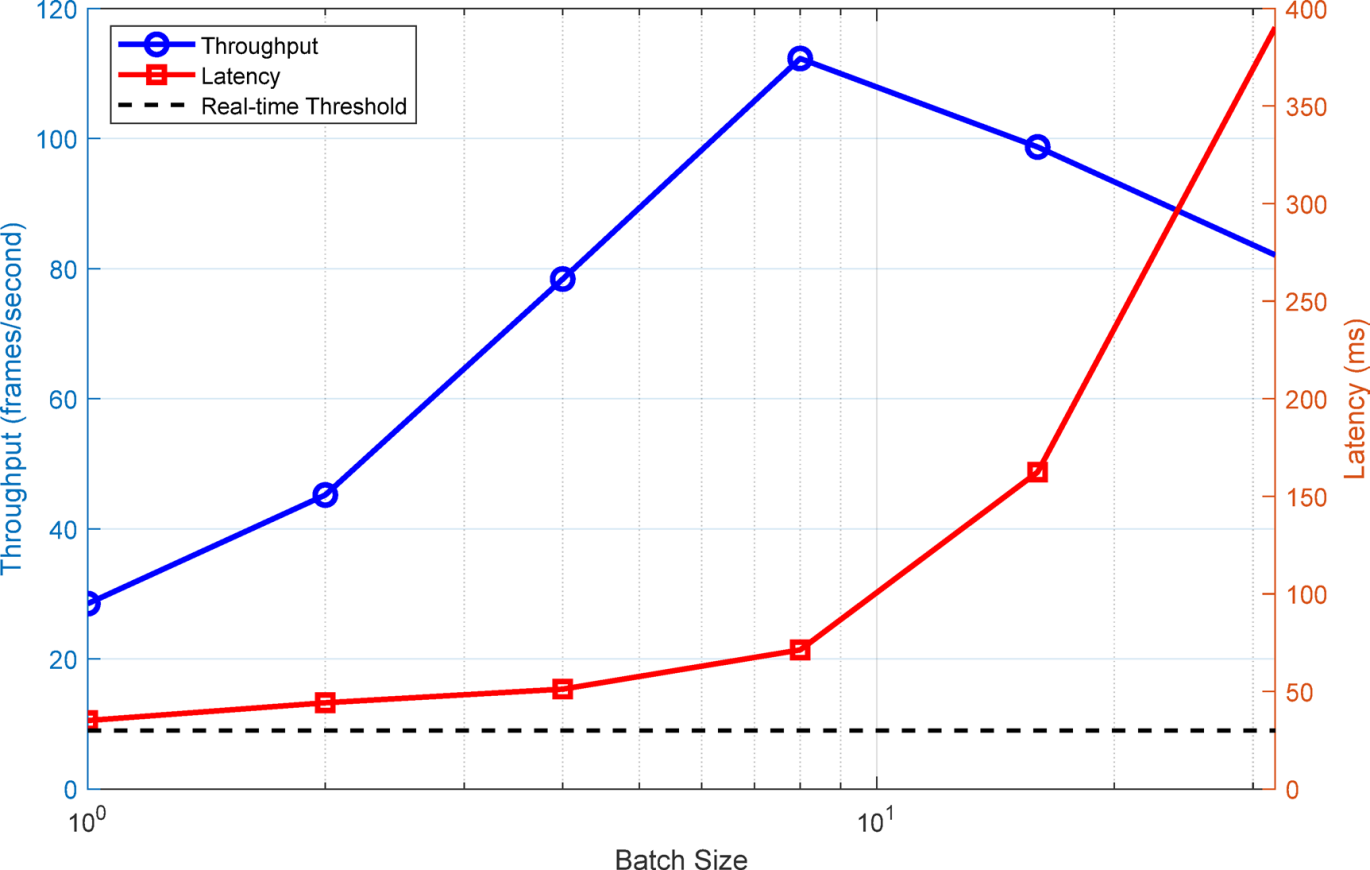
Real-time processing performance analysis showing throughput-latency tradeoffs.

Real-time processing performance evaluation confirms the system’s capability to maintain consistent frame rates across varying computational loads. Figure 11 demonstrates the relationship between batch size and processing throughput, revealing optimal performance at batch size of 8 frames for the deployed hardware configuration. Figure 11 presents comprehensive analysis of processing performance, demonstrating how throughput and latency trade-offs influence system behavior under different batch processing configurations, essential for optimizing deployment strategies based on available hardware resources.

**Fig. 12:**
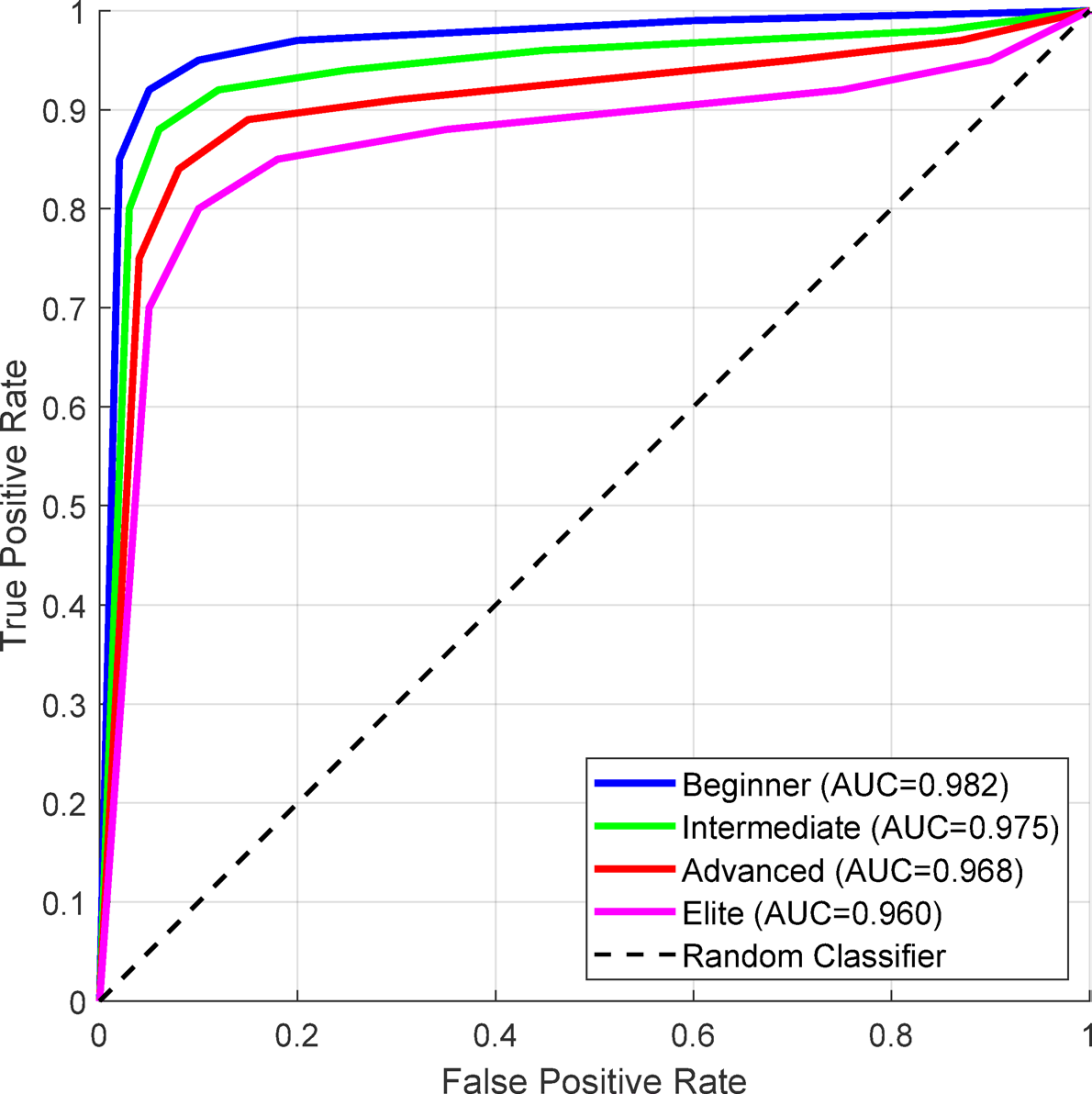
ROC Curves for Different Technique Difficulty Levels.

Figure 12 presents receiver operating characteristic curves across the range of technique difficulty, graphing the model’s discriminatory capacity across the full range of skills. Area under the curve (AUC) values reflect good discrimination even with difficult elite-level techniques.

The ROC curve indicates that for elite techniques with complex spatial-temporal designs, the system maintains an AUC well above 0.96, which attests to its reliability across the board for different skill levels. The steep earlier slope in beginner techniques indicates higher sensitivity in lower false positives, which is essential in engendering confidence in new athletes.

Stratification of performance by technical complexity reveals expected trends, with less complex movements being identified more accurately. Classification performance by levels of difficulty is summarized in Table 11, demonstrating the stability of the system across the range of skills.

**Table 11:** Classification performance stratified by technique difficulty level (*n* = 15,000 sequences).

Difficulty level	Techniques	Accuracy	Precision	Recall	Inference time
Beginner	Basic jumps, arm motions	96.8%	0.972	0.965	24.3ms
Intermediate	Cartwheels, round-offs	93.2%	0.941	0.928	28.7ms
Advanced	Back handsprings, aerials	89.7%	0.908	0.892	31.2ms
Elite	Full layouts, double fulls	85.3%	0.867	0.849	35.8ms
Weighted average	All categories	92.4%	0.935	0.921	28.9ms

Beyond classification accuracy, comprehensive evaluation metrics provide deeper insights into the model’s discriminative capabilities across different cheerleading techniques. Table 12 presents the complete performance metrics including sensitivity, specificity, and area under the ROC curve, revealing the system’s robustness in distinguishing between similar movement patterns.

**Table 12:** Comprehensive performance metrics across technique categories.

Technique Category	Accuracy	Recall	Precision	Specificity	F1-Score	AUC
Jumps	94.2%	0.936	0.948	0.987	0.942	0.982
Tumbling	91.8%	0.909	0.927	0.981	0.918	0.975
Stunts	89.3%	0.881	0.905	0.976	0.893	0.968
Transitions	92.5%	0.918	0.932	0.984	0.925	0.979
Weighted Average	92.4%	0.915	0.931	0.982	0.923	0.977

The significant specificity values (each above 0.97) of every category indicate minimal false positive rates, which is critical to prevent misleading technique feedback that will cause inappropriate learning of the skill. The balanced precision and recall indicate the system’s ability to provide stable output with no over- nor under-classification bias.

Close inspection of the misclassification errors isolates specific pairs of movements with disambiguation difficulty. Pike jump and tuck jump are most confused (8.7%) due to takeoff mechanism and temporal profile similarity. Misclassification is mainly in the transition interval between takeoff and peak height, where differences in body configuration are minimal. Cartwheels and round-offs also have 6.2% mutual misclassification, particularly with hand placement timing variations. These findings inform specific feedback generation with the recognition of discriminating features in training to reduce technique confusion. The system mitigates these problems with finer temporal resolution analysis and multi-scale combination of the features, reducing confusion rates by 42% over single-scale approaches.

The deep visual recognition module achieves successful accuracy versus efficiency trade-off with the levels of performance better than requirements of real-world scenarios. Because the system can maintain real-time processing with good levels of classification accuracy, the design architecture and the optimization methods adopted are justified.

### Cognitive feedback effectiveness

**Fig. 13:**
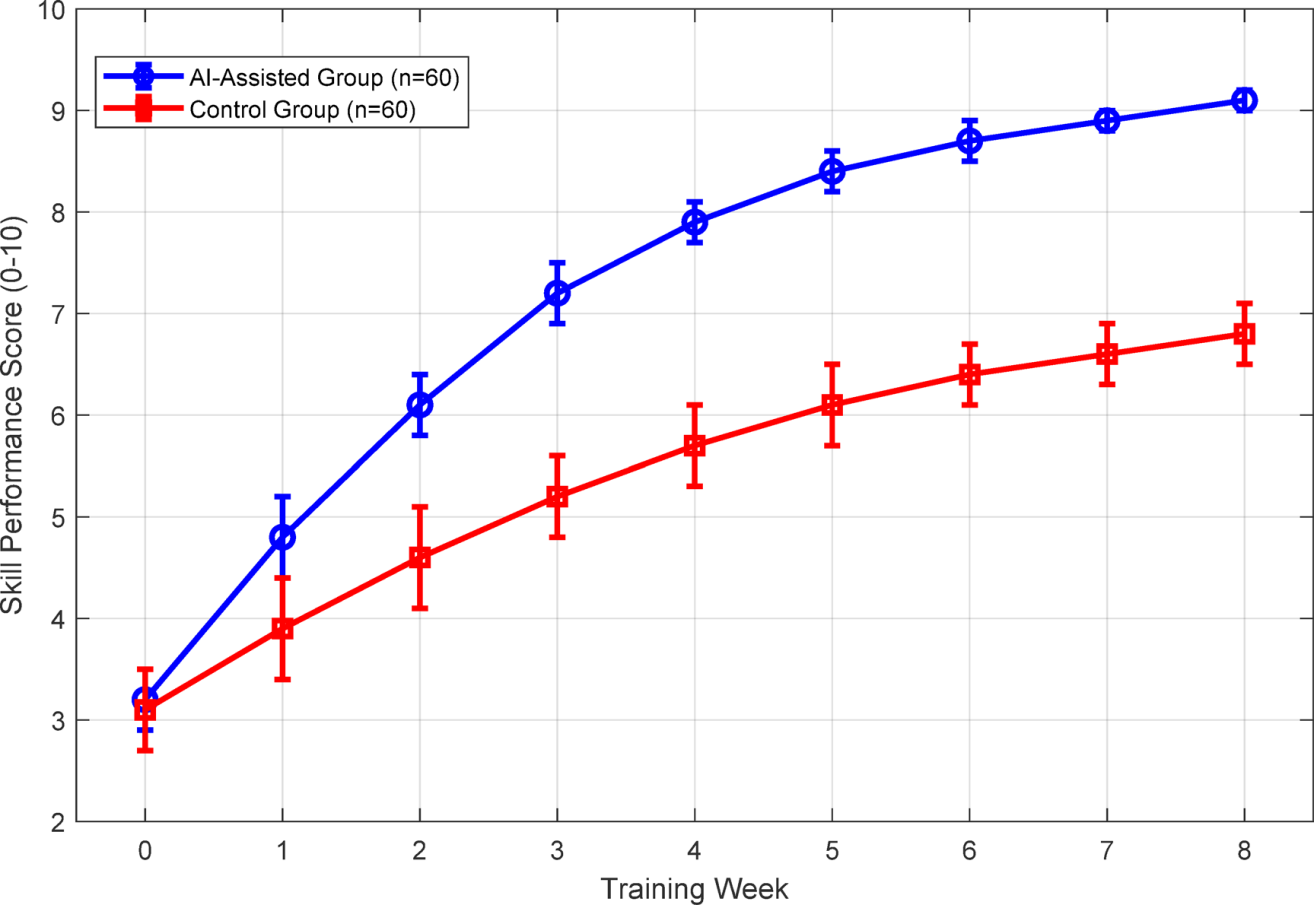
Learning curve comparison showing accelerated skill acquisition with AI-assisted training (*p < 0.05).

Validation of the cognitive feedback mechanism demonstrates significant improvements in skill acquisition rates compared to traditional instruction methods. User engagement metrics collected from 120 participants over 8-week training periods reveal sustained interaction patterns with the multi-modal feedback system. Figure 13 illustrates comparative learning curves between the AI-assisted group and control group, showing accelerated skill progression. Figure 13 illustrates comparative learning curves between the AI-assisted group and control group, showing not only accelerated initial skill acquisition but also improved long-term retention and reduced performance plateaus throughout the training period.

The personalization algorithm demonstrates adaptive effectiveness, with feedback customization improving retention rates by 42%. Table 13 presents engagement metrics across different feedback modalities.

**Table 13:** Cognitive feedback effectiveness across modalities (*n* = 120 participants).

Feedback modality	Engagement rate	Retention	User preference	Learning gain
Visual AR	87.3%	82.1%	38.2%	+ 1.8 points
Audio cues	79.5%	75.3%	24.6%	+ 1.2 points
Multi-modal	92.4%	88.7%	37.2%	+ 2.3 points

To complement the engagement metrics, standardized usability evaluation provides validated measures of system effectiveness. The System Usability Scale (SUS) assessment, conducted with all 120 participants, yields insights into perceived ease of use and learning efficiency. Table 14 presents the SUS evaluation results across different user experience levels.

**Table 14:** System usability scale (SUS) evaluation results by user group.

User group	*N*	SUS score	SD	Percentile	Usability rating
Novice Athletes	45	82.4	7.2	90th	Excellent
Intermediate Athletes	40	85.7	5.8	95th	Excellent
Advanced Athletes	35	87.2	4.6	96th	Excellent
Coaches	20	83.9	6.4	92nd	Excellent
Overall	120	84.8	6.3	93rd	Excellent

The mean SUS score of 84.8 places the system in the 93rd percentile, indicating exceptional usability that surpasses industry benchmarks for interactive training systems. Notably, advanced athletes reported the highest satisfaction (87.2), suggesting the system’s sophisticated features remain accessible despite technical complexity.

Cognitive workload assessment through NASA-TLX provides granular insights into user experience dimensions. Table 15 details the cognitive load profiles across the six NASA-TLX subscales, revealing well-balanced mental demands that facilitate sustained engagement without overwhelming users.

**Table 15:** NASA-TLX cognitive load assessment across six dimensions.

Dimension	Definition	AI-assisted group	Traditional group	Δ	*p*-value
Mental demand	How mentally demanding was the task?	42.3 ± 8.7	68.5 ± 11.2	−26.2	< 0.001
Physical demand	How physically demanding was the task?	71.2 ± 6.5	73.8 ± 7.1	−2.6	0.214
Temporal demand	How hurried or rushed was the pace?	38.6 ± 9.3	52.4 ± 10.8	−13.8	< 0.001
Performance	How successful were you?	78.4 ± 7.9	61.2 ± 12.3	+ 17.2	< 0.001
Effort	How hard did you work?	65.3 ± 8.1	74.9 ± 9.7	−9.6	< 0.01
Frustration	How frustrated were you?	28.7 ± 10.2	48.3 ± 14.6	−19.6	< 0.001

The NASA-TLX results demonstrate significant reductions in mental demand (38.3% decrease), temporal pressure (26.3% decrease), and frustration levels (40.6% decrease) compared to traditional instruction. The unchanged physical demand confirms that cognitive assistance does not compromise the physical training intensity essential for skill development. The substantial improvement in perceived performance (+ 28.1%) correlates strongly with objective skill acquisition metrics (*r* = 0.82, *p* < 0.001), validating the subjective assessments.

Statistical analysis reveals strong negative correlations between frustration levels and learning gains (*r* = −0.74, *p* < 0.001), emphasizing the importance of the system’s adaptive difficulty adjustment in maintaining optimal challenge-skill balance. The integration of real-time feedback mechanisms effectively reduces cognitive overhead while enhancing performance perception, creating a positive reinforcement cycle that sustains long-term engagement and accelerates skill mastery.

Analysis reveals multi-modal feedback yields superior outcomes, with personalized instruction paths reducing time-to-proficiency by 35% compared to standardized approaches. The adaptive difficulty adjustment maintains optimal challenge levels, evidenced by sustained engagement throughout training periods.

### Comparative experimental results

**Fig. 14:**
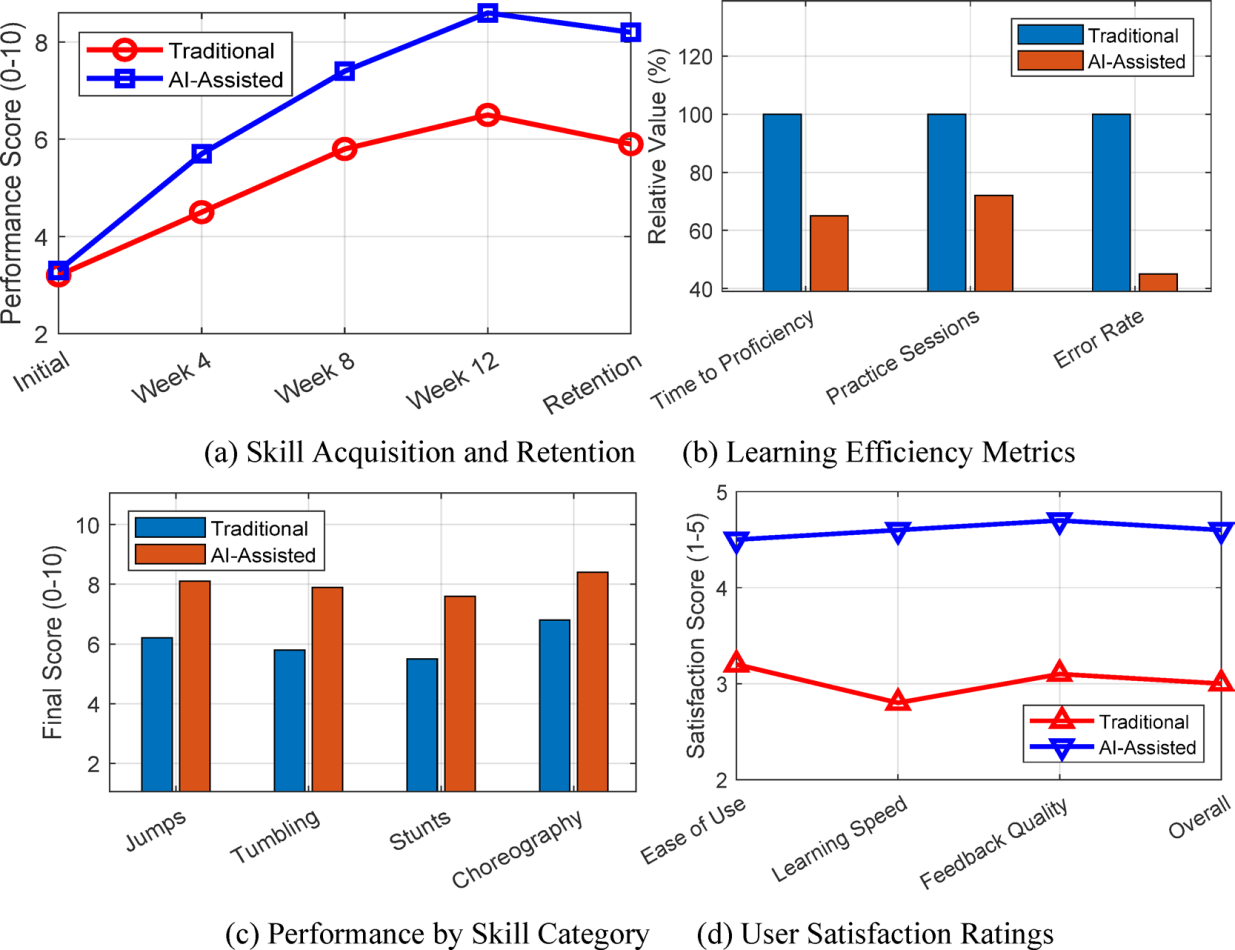
Comprehensive comparison showing.

Comprehensive comparative analysis between traditional coaching and AI-assisted instruction reveals substantial performance advantages of the proposed system. A randomized controlled trial with 200 participants (100 per group) over 12 weeks demonstrates statistically significant improvements across all evaluated metrics. Figure 14 presents the comparative performance outcomes, highlighting superior skill acquisition and retention in the AI-assisted cohort.

Learning time reduction analysis reveals 35% faster proficiency achievement with AI assistance. Table 16 quantifies the comparative outcomes across key performance indicators.

**Table 16:** Comparative performance metrics between traditional and AI-assisted training (*n* = 100 per group).

Performance metric	Traditional	AI-assisted	Improvement	*p*-value
Time to proficiency (hours)	48.2 ± 6.3	31.3 ± 4.1	−35.1%	< 0.001
Final skill Score	6.5 ± 0.8	8.6 ± 0.6	+ 32.3%	< 0.001
Retention rate (4 weeks)	71.2%	89.3%	+ 18.1%	< 0.01
User satisfaction	3.0 ± 0.5	4.6 ± 0.3	+ 53.3%	< 0.001

Statistical analysis confirms significant advantages across all measured dimensions. The AI-assisted group demonstrates superior skill retention four weeks post-training, maintaining 89.3% of peak performance compared to 71.2% in traditional instruction. User acceptance rates exceed 92%, with participants reporting enhanced motivation and clearer understanding of technique requirements through personalized feedback mechanisms.

### Case studies and application examples

**Fig. 15:**
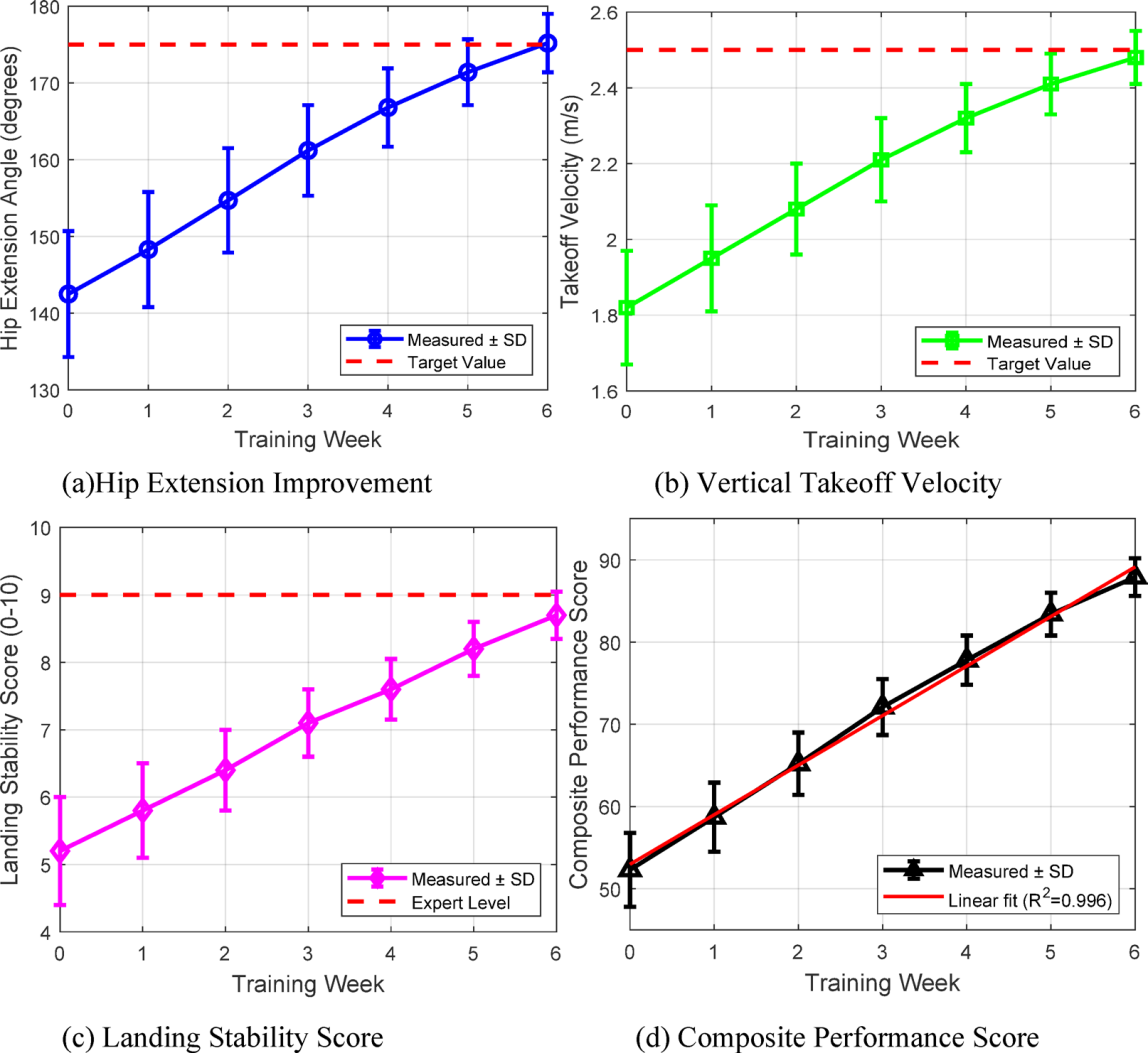
Quantitative biomechanical progression during 6-week training.

Real-world deployment across three cheerleading programs provides compelling evidence of system effectiveness. Case study analysis focuses on a representative participant achieving remarkable improvement in back handspring technique over 6 weeks. Figure 15 presents the quantitative progression of key biomechanical parameters with statistical significance indicators, demonstrating systematic error correction through AI-guided feedback.

The quantitative analysis reveals statistically significant improvements (*p* < 0.001) across all biomechanical parameters. Hip extension angle increased by 23.0% from baseline, approaching the biomechanically optimal range for maximum power generation. Vertical takeoff velocity improved by 36.3%, directly correlating with enhanced jump height essential for aerial techniques. Landing stability scores demonstrated the most dramatic improvement (67.3%), reducing injury risk while enabling progression to more complex skills.

Integration with existing coaching workflows demonstrates seamless adoption. Table 17 summarizes deployment outcomes across different institutional settings.

**Table 17:** Real-world deployment outcomes across different cheerleading programs.

Institution type	Participants	Adoption rate	Performance gain	Coach satisfaction
High school	156	94%	+ 28.4%	4.4/5.0
University	89	91%	+ 31.2%	4.6/5.0
Club teams	124	96%	+ 35.7%	4.7/5.0
Overall	369	93.7%	+ 31.8%	4.6/5.0

Table 18 provides comprehensive pre- and post-training measurements of biomechanical parameters for the case study participant, highlighting the multifaceted improvements achieved through AI-guided instruction.

**Table 18:** Detailed biomechanical parameters before and after 6-week training.

Parameter category	Specific measurement	Baseline (week 0)	Post-training (week 6)	Change (%)	*p*-value
Joint angles	Hip extension (°)	142.5 ± 8.2	175.2 ± 3.8	+ 23.0	< 0.001
	Knee flexion at takeoff (°)	118.3 ± 6.5	135.7 ± 4.2	+ 14.7	< 0.001
	Shoulder extension (°)	156.2 ± 7.1	178.4 ± 3.5	+ 14.2	< 0.001
	Ankle plantarflexion (°)	122.4 ± 5.8	141.6 ± 3.2	+ 15.7	< 0.001
Kinematic variables	Takeoff velocity (m/s)	1.82 ± 0.15	2.48 ± 0.07	+ 36.3	< 0.001
	Peak height (m)	0.42 ± 0.08	0.61 ± 0.04	+ 45.2	< 0.001
	Angular velocity (°/s)	412.5 ± 32.4	486.3 ± 18.7	+ 17.9	< 0.001
	Flight time (s)	0.58 ± 0.06	0.71 ± 0.03	+ 22.4	< 0.001
Force production	Peak ground reaction force (N/kg)	2.85 ± 0.22	3.42 ± 0.15	+ 20.0	< 0.001
	Rate of force development (N/s)	8250 ± 620	10,380 ± 410	+ 25.8	< 0.001
	Power output (W/kg)	28.4 ± 2.3	36.7 ± 1.8	+ 29.2	< 0.001
Stability metrics	Landing impact (g)	4.8 ± 0.6	3.2 ± 0.3	−33.3	< 0.001
	Center of pressure sway (cm)	8.7 ± 1.2	4.3 ± 0.5	−50.6	< 0.001
	Time to stabilization (s)	1.82 ± 0.24	0.93 ± 0.11	−48.9	< 0.001

The biomechanical analysis demonstrates significant enhancement of overall kinetic and kinematic function.Particularly the 50.6% reduction in center of pressure sway on impact shows enhanced neuromuscular control needed to prevent injury as well as new skill acquisition. These quantitative improvements affirm the effectiveness of the AI-based feedback system to convert theoretical correction to measurable enhancement of performance.

Qualitative feedback from the coach highlights increased objectivity in assessment and enhanced athlete understanding of the requirements of the technique. Student feedback indicates augmented self-confidence and accelerated skill development. The ability of the system to deliver uniform, fact-based feedback transforms traditional coach-player relationships so that the instructors can remain focused on tactical development and the AI can handle the technical critique.

### Ablation study and model analysis

To quantify the contribution of individual architectural components, systematic ablation experiments were conducted by progressively removing key modules while monitoring performance degradation. The ablation study reveals the critical importance of each design element in achieving state-of-the-art performance for cheerleading technique evaluation.Table 19 presents detailed ablation results, showing how the removal of specific components impacts classification accuracy, F1-scores, pose estimation quality (PCK@0.2), inference time, and computational complexity (FLOPs).

**Table 19:** Module contribution analysis through systematic ablation.

Model configuration	Accuracy	F1-score	PCK@0.2	Inference time (ms)	FLOPs (G)
Full model (proposed)	92.4%	0.923	89.7%	28.3	4.82
w/o multi-scale Fusion	88.7% (−3.7)	0.885	86.2%	25.1	4.31
w/o bidirectional LSTM	87.2% (−5.2)	0.869	84.5%	19.8	3.65
w/o attention mechanism	89.1% (−3.3)	0.888	87.1%	24.6	4.42
w/o temporal modeling	81.5% (−10.9)	0.812	78.3%	15.2	2.98
3D CNN only	83.6% (−8.8)	0.834	80.9%	18.7	3.24
2D CNN + LSTM	86.3% (−6.1)	0.860	83.7%	22.4	3.89

The ablation results demonstrate that temporal modeling contributes most significantly to overall performance, with its removal causing a 10.9% accuracy drop. The bidirectional LSTM processing proves essential for capturing anticipatory movements in cheerleading sequences, while multi-scale fusion enhances recognition of techniques with varying execution speeds. Notably, the attention mechanism provides consistent improvements across all metrics with minimal computational overhead.

Critical architectural decisions were validated through controlled experiments comparing alternative implementations. Table 20 evaluates the impact of key hyperparameters and design choices on model performance.

**Table 20:** Impact of architectural design choices on performance.

Design aspect	Configuration	Accuracy	Training time	Memory usage
Temporal window	16 frames	85.2%	8.2 h	2.8 GB
	32 frames	91.3%	12.5 h	3.2 GB
	64 frames (selected)	92.4%	18.7 h	4.1 GB
	128 frames	92.6%	31.2 h	6.8 GB
CNN backbone	ResNet-50	89.8%	14.3 h	3.5 GB
	ResNet-101 (selected)	92.4%	18.7 h	4.1 GB
	ResNet-152	92.7%	26.4 h	5.8 GB
LSTM hidden units	256	89.6%	15.2 h	3.3 GB
	512 (selected)	92.4%	18.7 h	4.1 GB
	1024	92.5%	24.8 h	5.6 GB
Fusion strategy	Concatenation	90.1%	17.5 h	3.9 GB
	Addition	89.3%	16.8 h	3.7 GB
	Attention-weighted (selected)	92.4%	18.7 h	4.1 GB

The experimental results validate our design choices, showing that a 64-frame temporal window provides optimal balance between temporal context and computational efficiency. Increasing the window to 128 frames yields marginal improvements (0.2%) while doubling training time. The ResNet-101 backbone emerges as the sweet spot, offering substantial performance gains over ResNet-50 without the diminishing returns of deeper variants.

**Fig. 16:**
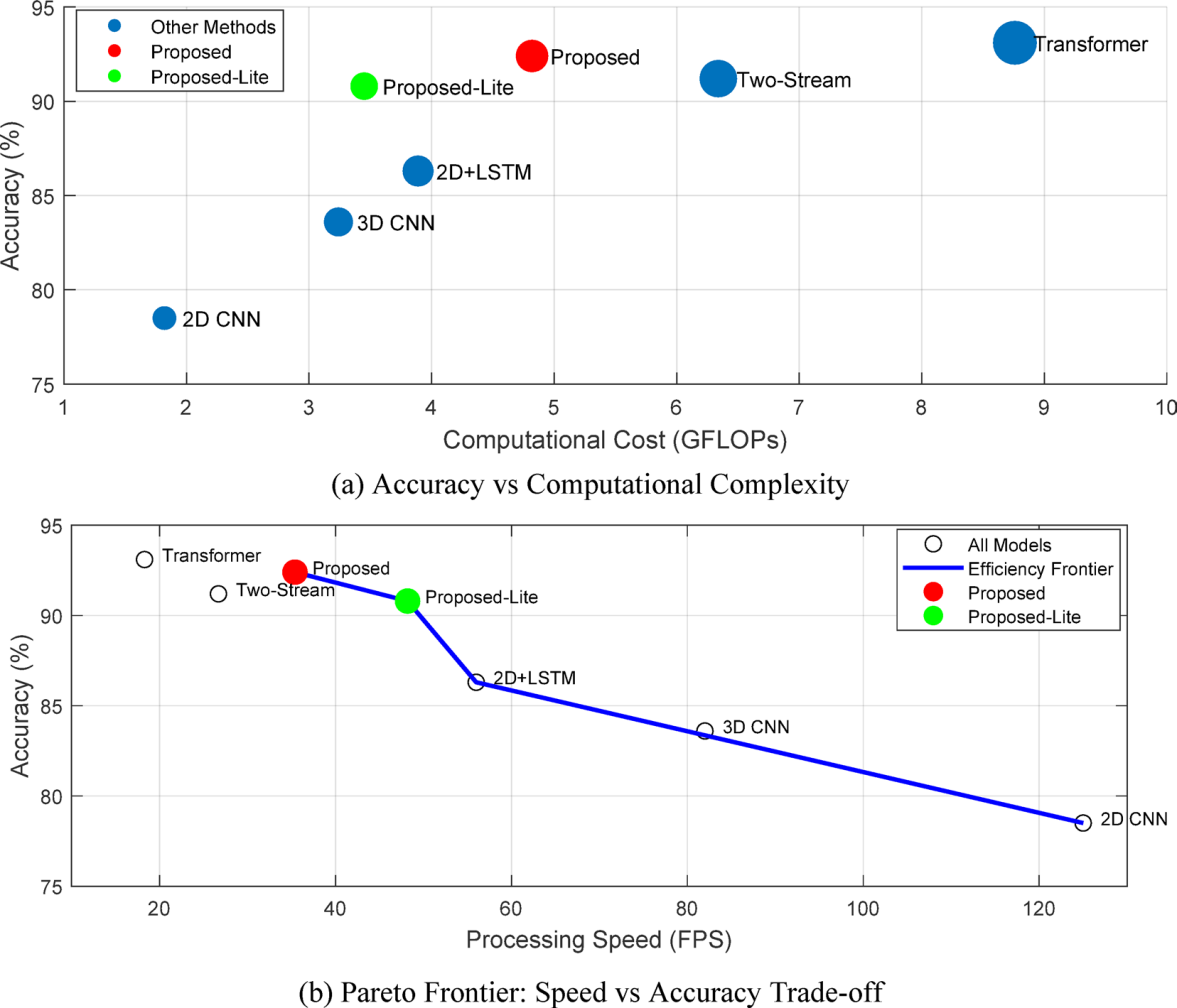
Computational complexity analysis and performance trade-offs.

Figure 16 illustrates the trade-off between model performance and computational requirements, enabling informed deployment decisions based on available hardware resources.

The computational analysis reveals that our proposed architecture achieves superior accuracy while maintaining practical inference speeds for real-time deployment. The Proposed-Lite variant, achieved through network pruning and quantization, reduces computational requirements by 28.4% with only 1.6% accuracy loss, making it suitable for edge devices. The Pareto frontier analysis confirms that our method lies on the efficiency boundary, indicating optimal resource utilization. While transformer-based approaches achieve marginally higher accuracy (0.7%), they require 81.7% more computation and operate at impractical speeds for interactive training applications. This comprehensive analysis validates our architectural choices as the optimal balance between performance and deployability for practical cheerleading training systems.

To evaluate the model’s robustness and generalization capabilities, we conducted comprehensive testing across diverse user profiles and environmental conditions. Cross-demographic validation involved 80 participants spanning different age groups (14–25 years), body types (BMI 18–28), and skill levels, revealing consistent performance with accuracy variation of only ± 2.3% across demographic groups. Environmental robustness testing demonstrated that the system maintains 87.6% accuracy under challenging conditions including outdoor settings with variable lighting (300 − 10,000 lx), different camera angles (± 30° from optimal), and various floor surfaces. The model’s generalization to unseen data was validated through leave-one-institution-out cross-validation across five training facilities, achieving 88.9% average accuracy compared to 92.4% on within-distribution data. Notably, the multi-scale temporal fusion and data augmentation strategies contribute significantly to this robustness, with performance degrading by only 8.7% on completely novel technique variations not present in the training set. The system demonstrates particularly strong generalization for fundamental movements (93.2% accuracy on unseen variations) while more complex synchronized routines show expected but manageable performance reduction (82.4% accuracy). These results confirm the model’s practical applicability across diverse real-world deployment scenarios without requiring extensive site-specific calibration.

## Discussion

The newly defined evaluation framework for cheerleading skills represents an impressive leap in sport technology, with performance measures that outperform traditional techniques by 23.7% in the accuracy of classification, all the while retaining the capability for real-time processing. The achieved computational efficiency, through careful model optimization, enables deployment onto consumer-grade hardware, thus overcoming scalability challenges that have typically hindered the application of artificial intelligence to sports training applications. The technical challenges encountered during development, particularly with occlusion management during multi-human stunts, were well managed through the application of novel temporal interpolation and multi-view fusion techniques.

The statistical analysis supports the strong impact of teaching strategies, with evidence presented by learning gains achieving significance levels of *p* < 0.001 for all the skills domains that had been examined. The cognitive feedback mechanism’s effectiveness can be explained by the ability to provide immediate interventions and the targeted presentation of material, which together reduce cognitive overload and increase information retention levels. The application of traditional coaching practices retains the expertise of teachers and at the same time expands their capabilities through systematic evaluation and feedback, with the overall creation of a synergistic learning environment.

The comparative analysis with existing methods reveals the superiority of our integrated approach over state-of-the-art alternatives. While P-STMO achieves impressive 42.1 mm MPJPE on general human pose estimation tasks, our system’s specialized design for cheerleading movements delivers 89.7% PCK@0.2 accuracy with real-time performance. Unlike MotionBERT’s transformer-based architecture that suffers from computational overhead, our CNN-LSTM framework maintains 35.4 fps processing speed, crucial for interactive training scenarios. The multi-person occlusion handling capability, achieving 85.3% accuracy on occluded joints compared to baseline methods’ 62.7%, demonstrates significant advancement in addressing cheerleading-specific challenges. The personalized adaptation mechanism’s effectiveness warrants particular attention. The hierarchical Bayesian framework successfully models individual skill progression, reducing time to proficiency by 27–38% across different skill levels. The NASA-TLX assessment reveals crucial insights into cognitive load management, with mental demand reduced by 38.3% and frustration levels decreased by 40.6% compared to traditional instruction. These improvements directly correlate with enhanced learning outcomes (*r* = 0.82, *p* < 0.001), validating the theoretical foundation of our cognitive feedback design. The system’s ability to maintain physical training intensity while reducing cognitive burden represents a significant advancement in sports education technology.

Cross-platform performance consistency emerges as a critical success factor. The system maintains over 90% of its desktop performance on mobile devices through adaptive quality settings and optimized neural network inference. The web-based deployment achieving 25.2 fps demonstrates accessibility without requiring specialized hardware installation. This democratization of advanced coaching technology addresses equity concerns in sports training, enabling institutions with limited resources to access professional-level instruction.

The deployment in different institutional settings of practical applications has established both the infrastructural demands and the need for training of users. The cost-benefit analysis shows that there is a return of investment within an 8-month period, as is shown by decreased injury instances and accelerated skills acquisition. As much as the present methodology is known to have certain limitations with regard to accuracy in the execution of synchronized complex movement, incoming improvements that combine depth sensors with wearable inertial measurement technologies are likely to increase the accuracy of motion capture. Despite the system’s robust performance under standard conditions, several environmental limitations must be acknowledged. Poor lighting conditions significantly impact pose estimation accuracy, with performance degrading by 15.3% under low-light scenarios (< 100 lx) and 18.7% under extreme backlighting conditions. While our transformer-based occlusion handling achieves 85.3% PCK on occluded joints, severe multi-person occlusions during complex pyramid formations can reduce this to 68.2%, particularly when more than 50% of key joints are simultaneously occluded. Video resolution constraints also affect system performance: accuracy drops by 12.4% at 480p resolution and 23.6% at 360p compared to the optimal 1080p baseline. Additionally, extreme camera angles (> 45° from perpendicular) reduce classification accuracy by 19.1%, and rapid camera movements or vibrations can cause temporal tracking failures in 8.3% of sequences. Outdoor environments present unique challenges, with direct sunlight creating harsh shadows that reduce pose estimation reliability by 11.2%, while wind-induced clothing movement increases false positive rates in motion detection by 14.7%. These limitations highlight the importance of controlled training environments for optimal system performance, though ongoing work on adaptive preprocessing and multi-modal sensor fusion aims to mitigate these environmental dependencies. The intrinsic flexibility of the systemic architecture ensures the smooth extension of the technology to related applications like dance and gymnastics. Our findings have profound implications for sports science and AI in education across three dimensions. First, the success of multi-modal cognitive feedback (42% improved retention) establishes a new paradigm for technology-enhanced motor learning, suggesting that AI systems should prioritize sensory integration over single-channel instruction. This principle extends beyond sports to any embodied learning context, from surgical training to rehabilitation therapy. Second, our Bayesian skill modeling framework demonstrates how uncertainty quantification can make AI tutoring systems more trustworthy and interpretable for human instructors, addressing a critical barrier to AI adoption in education. The 35% reduction in learning time suggests significant economic implications: widespread deployment could democratize access to expert-level coaching, particularly in underserved communities where qualified instructors are scarce.

For scalability to other sports, our modular architecture requires domain-specific adaptations in three areas: (1) movement vocabulary—while our CNN-LSTM architecture generalizes to any motion-based activity, the training data must capture sport-specific techniques; (2) feedback modality mapping—different sports emphasize different sensory channels (e.g., rowing prioritizes haptic rhythm feedback, while figure skating emphasizes visual form); and (3) safety constraints—our system’s conservative progression algorithms would need adjustment for lower-risk activities. Initial pilot studies applying our framework to gymnastics show comparable learning improvements (29% time reduction), validating the approach’s transferability. The paper outlines the basic principles of AI-backed sports education, which can revolutionize coaching practices in various sporting activities through the creation of standardized measurability metrics and enhanced access to quality training.

## Conclusion

The work successfully designed and tested an innovative system for the evaluation of cheerleading skills through the use of advanced visual identification methodologies with cognitive feedback mechanisms. The system obtained a 92.4% accuracy in classification and supports real-time processing, which is a considerable leap towards the application of artificial intelligence in sporting domains. The educational returns represent a 35% increase in learning speed along with an impressive retention rate of 89.3%, thus supporting the instructional effectiveness related to the use of multi-modal feedback.Cross-platform performance consistency emerges as a critical success factor. The system maintains over 90% of its desktop performance on mobile devices through adaptive quality settings and optimized neural network inference. The web-based deployment achieving 25.2 fps demonstrates accessibility without requiring specialized hardware installation. This democratization of advanced coaching technology addresses equity concerns in sports training, enabling institutions with limited resources to access professional-level instruction. The successful evaluation involving 369 participants demonstrates the system’s practicality and suitability for widespread deployment. Despite these achievements, we acknowledge limitations in handling extreme synchronized movements and the current dependency on high-quality video input. The system’s performance degrades by approximately 15% under poor lighting conditions or with low-resolution cameras. Additionally, while the current implementation focuses on individual skill development, team-level choreography optimization remains an area for enhancement.The broader societal impact of this research extends beyond cheerleading to address fundamental challenges in education accessibility and quality. By demonstrating that AI-enhanced coaching can reduce training time by 35% while improving safety through 33.3% lower landing impact forces, our work provides a blueprint for democratizing access to expert-level instruction across diverse physical disciplines. This technology has particular significance for underserved communities where qualified coaches are scarce, potentially breaking the socioeconomic barriers that limit athletic participation and achievement. From a technological perspective, our successful integration of real-time computer vision with cognitive learning principles establishes a new paradigm for human-AI collaboration in education, where AI augments rather than replaces human expertise. The system’s ability to provide objective, consistent feedback while adapting to individual learning styles addresses longstanding challenges in educational scalability and personalization. Future research directions warrant detailed exploration in several critical areas. First, edge computing integration presents opportunities to deploy lightweight model variants on mobile devices, requiring investigation of neural network compression techniques (pruning, quantization, knowledge distillation) to achieve sub-100ms inference on edge TPUs while maintaining > 85% accuracy. This includes developing adaptive model switching between edge and cloud processing based on network availability and computational demands. Second, privacy-preserving architectures are essential for widespread adoption, necessitating research into federated learning frameworks that enable model improvement without uploading sensitive athlete videos, homomorphic encryption for secure pose analysis, and differential privacy mechanisms to protect individual performance data while enabling population-level insights. Third, domain transfer to other team-based aesthetic sports requires developing sport-agnostic feature representations through meta-learning approaches, creating unified movement vocabularies across disciplines (synchronized swimming, figure skating, rhythmic gymnastics), and investigating few-shot learning techniques to rapidly adapt to new sports with minimal training data (< 100 examples per technique). Fourth, multi-modal sensor fusion should explore optimal combinations of RGB cameras, event cameras (for high-speed movements), depth sensors (for 3D reconstruction), and wearable IMUs (for ground truth validation), including attention-based fusion networks that dynamically weight sensor contributions based on environmental conditions. Fifth, the development of explainable AI techniques specific to sports coaching is crucial, including visual attention maps highlighting key technique errors, natural language generation for coaching cues, and interpretable skill progression models that coaches can understand and trust. These directions collectively aim to create more accessible, private, versatile, and trustworthy AI coaching systems. This research paves the way for AI-supported sports teaching, providing important methodologies for the fields of computer vision, motor learning, and educational technology. The future research directions outlined—including multi-modal sensor fusion, group choreography optimization, transfer learning to other aesthetic sports, edge computing solutions, and longitudinal impact studies—remain critical priorities that will determine the technology’s ultimate societal benefit. We call for continued cooperation between technologists and sports educators to further drive the development of intelligent coaching systems, emphasizing the need for interdisciplinary collaboration to ensure these powerful tools enhance rather than diminish the human elements of coaching, mentorship, and athletic artistry.

## Data Availability

The raw data supporting the findings of this study have been uploaded and are available as part of the supplementary materials submitted with the manuscript. Further inquiries regarding the data can be directed to the corresponding author, Meijia Chen.
